# Chimeric antigen receptor‐modified human regulatory T cells that constitutively express IL‐10 maintain their phenotype and are potently suppressive

**DOI:** 10.1002/eji.202048934

**Published:** 2021-08-08

**Authors:** Yasmin R. Mohseni, Adeel Saleem, Sim L. Tung, Caroline Dudreuilh, Cameron Lang, Qi Peng, Alessia Volpe, George Adigbli, Amy Cross, Joanna Hester, Farzin Farzaneh, Cristiano Scotta, Robert I. Lechler, Fadi Issa, Gilbert O. Fruhwirth, Giovanna Lombardi

**Affiliations:** ^1^ MRC Centre for Transplantation Immunology School of Immunology and Microbial Sciences, King's College London London UK; ^2^ Imaging Therapies and Cancer Group Comprehensive Cancer Centre, School of Cancer and Pharmaceutical Studies, King's College London London UK; ^3^ Department of Haematology and Precision Medicine Kings College Hospital London UK; ^4^ Transplantation Research & Immunology Group, Nuffield Department of Surgical Sciences University of Oxford, Oxford, UK; ^5^ Department of Haematological Medicine School of Cancer and Pharmaceutical Studies, King's College London London UK

**Keywords:** Cell therapy, Chimeric antigen receptor, IL‐10, Regulatory T cell, Suppression

## Abstract

Clinical trials of Treg therapy in transplantation are currently entering phases IIa and IIb, with the majority of these employing polyclonal Treg populations that harbor a broad specificity. Enhancing Treg specificity is possible with the use of chimeric antigen receptors (CARs), which can be customized to respond to a specific human leukocyte antigen (HLA). In this study, we build on our previous work in the development of HLA‐A2 CAR‐Tregs by further equipping cells with the constitutive expression of interleukin 10 (IL‐10) and an imaging reporter as additional payloads. Cells were engineered to express combinations of these domains and assessed for phenotype and function. Cells expressing the full construct maintained a stable phenotype after transduction, were specifically activated by HLA‐A2, and suppressed alloresponses potently. The addition of IL‐10 provided an additional advantage to suppressive capacity. This study therefore provides an important proof‐of‐principle for this cell engineering approach for next‐generation Treg therapy in transplantation.

## Graphical abstract

Chimeric antigen receptor (CAR) constructs can be modified to incorporate elements for enhanced activity. Here, human Tregs were modified to express an anti‐HLA‐A2 CAR as well as IL‐10 and an imaging reporter. These cells maintained a stable Treg phenotype after lentiviral transduction, were specifically activated by cells expressing HLA‐A2, and had enhanced in vitro suppressive potency compared with CAR Tregs without constitutive IL‐10 expression.

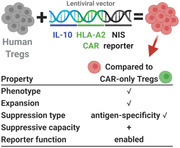

## Introduction

FOXP3^+^ regulatory T cells (Tregs) are a subset of CD4^+^ T cells that function to maintain self‐tolerance and prevent inappropriate immune activation [1, 2]. Tregs are currently under investigation as an adoptive cell‐based therapy to prevent transplant rejection and for the treatment of autoimmune diseases [3, 4]. Polyclonal Treg cellular therapy trials have shown promise in the prevention of graft‐versus‐host disease after allogeneic HSC transplantation [5, 6], as well as in the maintenance of C‐peptide levels in type 1 diabetes [7, 8]. We have completed two phase I/II clinical trials, the ONE Study (NCT02129881) and ThRIL (NCT02166177), both assessing the safety and feasibility of adoptive transfer of polyclonal Tregs [9, 10, 11, 12, 13, 14]. These trials have found Treg therapy to be safe, well tolerated, and with some early signs of efficacy [13, 15]. However, evidence indicates that donor‐specific Tregs are superior to polyclonal Tregs in pre‐clinical models of transplantation [16, 17, 18], with donor‐specificity achieved by culturing Tregs with allogeneic antigen‐presenting cells (APCs) or by transduction of Tregs with T‐cell receptors (TCR) specific for alloantigens [19].

Antigen specificity may also be conferred through the genetic engineering of Tregs to express chimeric antigen receptors (CARs). CARs are synthetic fusion proteins that comprise an extracellular antigen‐targeting domain, hinge and transmembrane domains, one or more intracellular costimulatory domains, and a TCR‐derived intracellular signaling domain [20, 21, 22]. CAR technology has the benefit of being customizable, from the target antigen to the signaling domains. Notably, CARs bypass major histocompatibility class (MHC) restriction. We and others have generated human Tregs expressing an HLA‐A2‐specific CAR and have shown that A2‐CAR‐Tregs are functionally superior in vitro and in vivo compared with polyclonal Tregs in a variety of humanized mouse models [23, 24, 25]. CAR engineering provides additional opportunities to produce cells that express other molecules as additional payloads. IL‐10 is an anti‐inflammatory cytokine produced by a wide range of both innate and adaptive immune cells that acts to limit inflammatory responses and prevent autoimmunity [26]. IL‐10 signals through activation of the Jak kinase Stat transcription factor pathway, and although Stat3 is indispensable for this process, both Stat1 and Stat 5 have also been shown to be relevant to its signaling. IL‐10 suppresses through the inhibition of proinflammatory cytokine secretion, as well as direct suppression of Th2 and Th17 cells and APCs [27, 28]. Tregs can express IL‐10 in vivo, but only after stimulation. The precise signals required for this are not yet clear but may be related to TGF‐β signaling [29]. Interestingly, IL‐10 can act in an autocrine fashion to further stimulate Tregs and enhance their function [30]. IL‐10 has also been shown to promote the generation of suppressive regulatory‐type 1 T cells (Tr1 cells) in vitro [31]. However, the effects of IL‐10 are complex and its regulation key to the eventual outcomes [27]. For example, in specific situations IL‐10 can promote the function of NK cells, CD8^+^ T cells, and B cells [32], resulting in enhanced anti‐tumor immune responses in some experimental settings [33]. These pleiotropic effects may explain the failures in clinical trials of direct IL‐10 administration in inflammatory bowel disease [32], and there is therefore an argument for a targeted IL‐10 approach to control for any aberrant pro‐inflammatory effects. In this context and to explore the potential role of IL‐10 as an additional CAR‐Treg payload, we built on our previously CAR‐Treg work [24] to examine the impact of co‐expression of IL‐10 in HLA‐A2 CAR Tregs.

## Results and discussion

### Generation of A2‐CAR‐Tregs co‐expressing IL‐10

**Figure 1:**
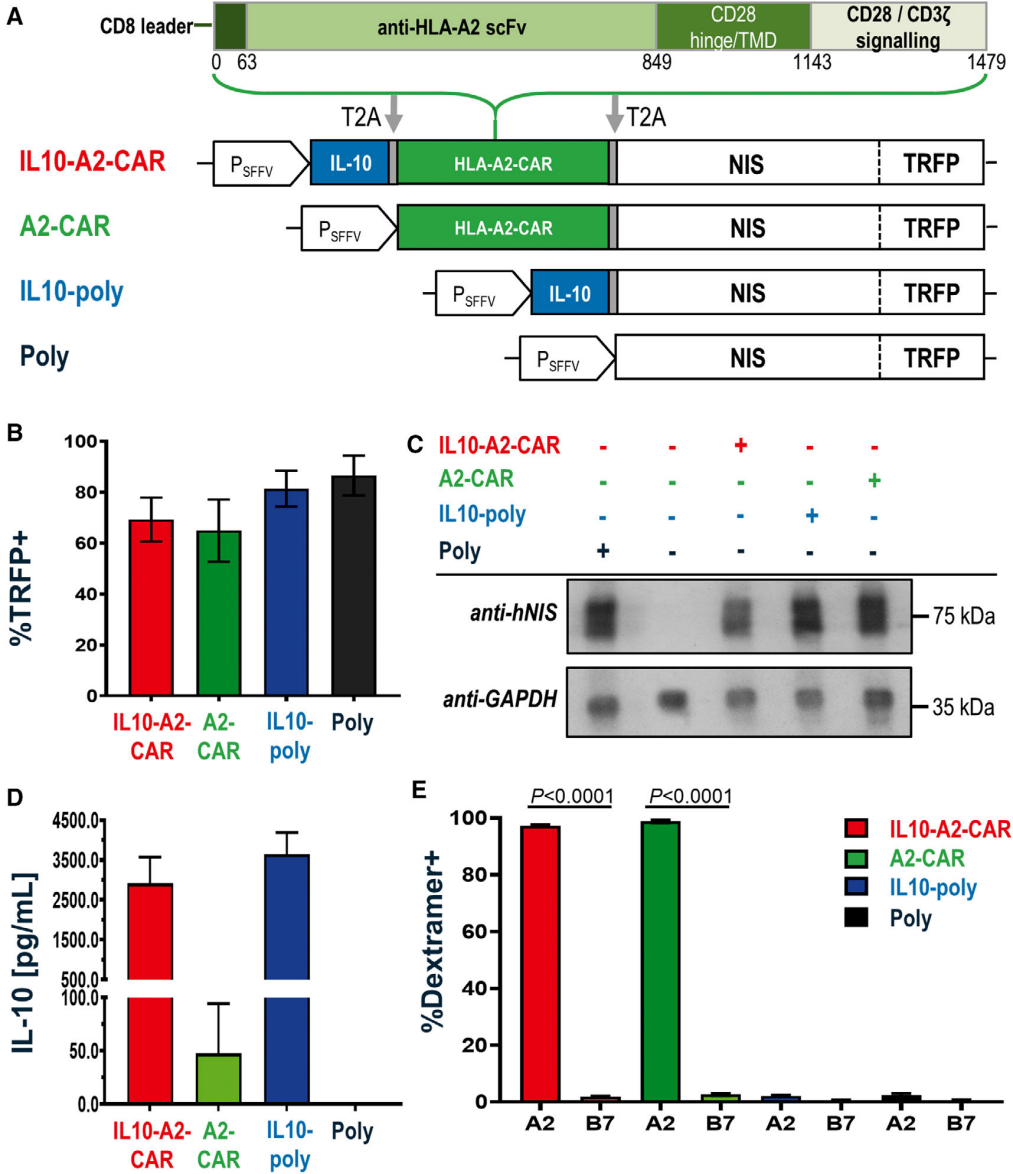
**Generation of different human Treg types. (A)** Cartoon of lentiviral expression constructs used in this study. Indicated elements are as follows: interleukin‐10 (IL‐10); CAR specific to the human HLA‐A2 antigen (HLA‐A2‐CAR); radionuclide‐fluorescence reporter formed of the sodium iodide symporter (NIS) fused to the red fluorescent protein TagRFP (NIS‐TRFP). All constructs were joined with T2A cleavage self‐cleavage sites and the cassette was driven by the spleen foci‐forming virus promoter (P_SFFV_). The total ORF lengths were 4.8, 4.2, 3, and 2.7 kb, respectively. Required regions in the lentiviral backbone including the P_SFFV_ promotor added 3.8 kb to each construct to form the complete gene transfer cassette. **(B)** Percentage of TRFP‐positive Tregs upon harvest at day 20 and measured by flow cytometry. **(C)** Representative immunoblot of indicated Tregs. Expected patterns for glycosylated and nonglycosylated NIS‐RFP were observed in transduced cells with GAPDH as a loading control. Representative data from the one of three experiments (same donor) are shown. See Supporting Information Fig. S11 for uncropped immunoblot. **(D)** IL‐10 analysis of Treg culture supernatant on day 20 by ELISA. **(E)** HLA‐A2‐specific and HLA‐B7 ("irrelevant") dextramers were used to quantify CAR surface expression on day 20 by flow cytometry. Data show means of *n =* 6 (B, E) or *n* = 3 (D) donors, with one donor per experiment; error bars are mean ± SEM. *p*‐Values calculated by comparing A2 and B7 conditions for each cell type using an unpaired Student's *t*‐test.

We engineered four expression cassettes containing (i) both IL‐10 and the HLA‐A2‐CAR (“IL10‐A2‐CAR”; open reading frame [ORF] size 4.85 kb, provirus size 8.67 kb), (ii) CAR alone (“A2‐CAR”), (iii) IL‐10 alone (“IL10‐poly”), and (iv) neither cassette (“Poly”) (Fig. 1A, Supporting Information Tables S1–S5). Notably, all constructs contained the established radionuclide‐fluorescence reporter NIS‐TRFP [34, 35] intended to streamline CAR‐Treg production and comparison (through its red fluorescence aiding flow cytometry/FACS) and enable future in vivo CAR‐Treg tracking. It is noteworthy that this fusion reporter has already been shown to not impact negatively on T cells [36]. Human Tregs were isolated and expanded in vitro as previously described (Supporting Information Fig. S1 and B; [9]). Transduction efficiencies were evaluated by NIS‐TRFP expression (Supporting Information Fig. S1C) before transduced cells were FACS sorted on day 10 and further expanded until day 20 (Fig. 1B, Supporting Information Fig. S1D). All further analyses refer to cells that were expanded for 10 days, FACS‐sorted, and expanded for further 10 days before indicated assays, which also involved gating on NIS‐TRFP‐positive and thus successfully transduced cells after expansion. In transduced Tregs, we also found the radionuclide reporter to be expressed and functional (Fig. 1C, Supporting Information Fig. S2). Notably, Treg lines transduced with an expression cassette encoding for IL‐10 secreted this cytokine at high levels (Fig. 1D). Moreover, those Treg lines transduced with vectors encoding for the A2‐CAR were confirmed to express the CAR by positive staining with an HLA‐A2 dextramer (Fig. 1E).

### Characterization of A2‐CAR‐Tregs co‐expressing IL‐10

**Figure 2:**
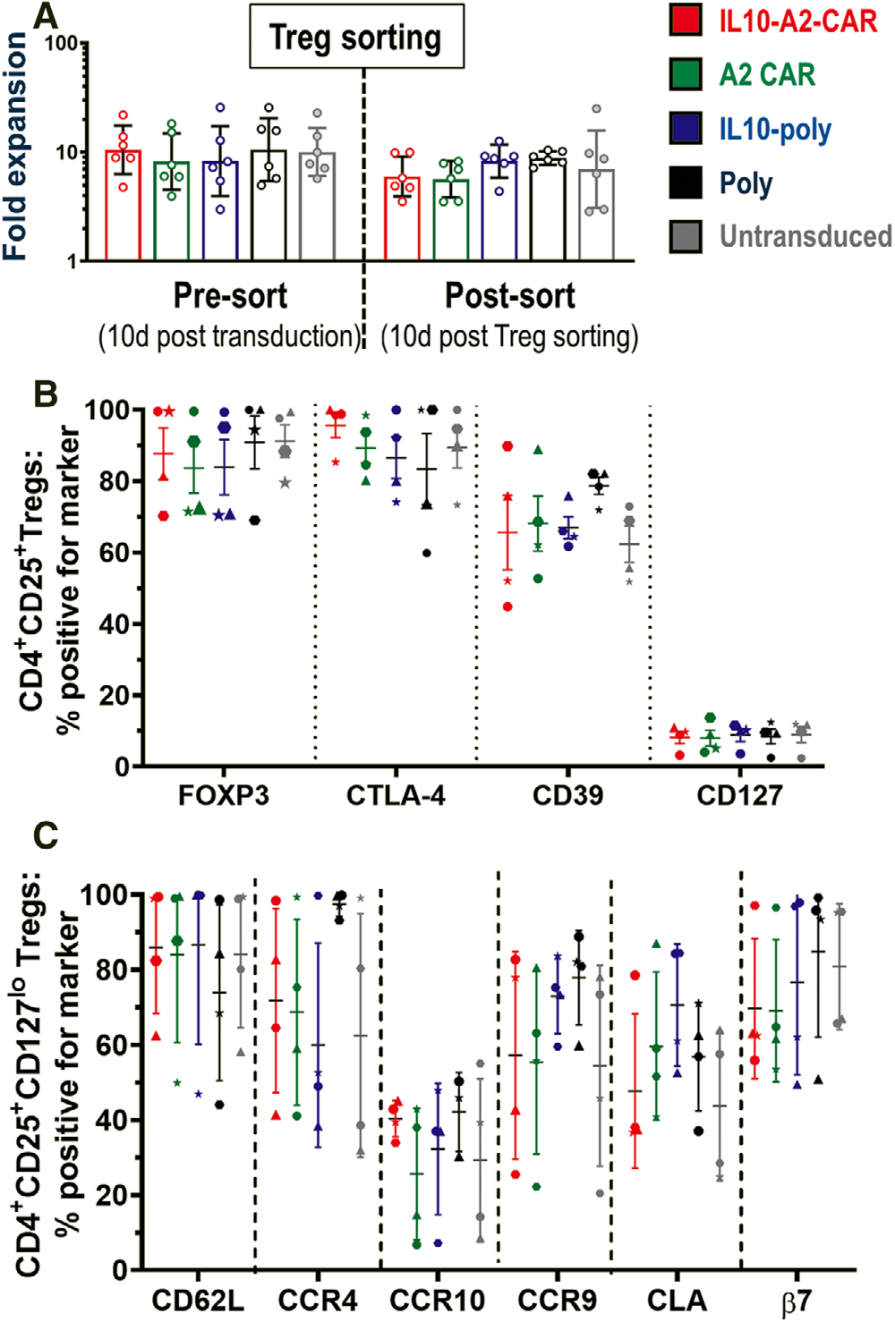
**Expansion capacity and phenotypes of differently transduced Tregs. (A) **Expansion capacity of indicated Treg types after transduction (days 0–10; left) and after FACS sorting (days 10–20; right) and compared to mock‐treated untransduced cells (gray). Geometric means and SD of *n* = 6 different Treg batches (different donors, one donor per experiment) are shown. No significant differences were found between Treg types neither before (*p* = 0.9840) nor after FACS sorting (*p* = 0.5445; both by one‐way ANOVA); however, expansion slowed across Treg types after FACS (*p* = 0.0340; two‐way ANOVA with Tukey's multiple comparison correction). **(B) **Different Treg types (colors as in (A)) were analyzed for expression of indicated markers. Cells were first gated on CD4^+^CD25^+^. **(C)**  Phenotypic marker analysis as in (B) but based on gating on CD4^+^CD25^+^CD127^lo^ cells (cf. CD127 marker in (B)). For both (B) and (C), cells were analyzed on day 20. Each individual data point belongs to one Treg batch/donor (symbol shapes identify donors). Error bars are mean ± SD from *n* = 4 different donors (one donor per experiment); no significant differences in marker expression between Treg types were found by one‐way ANOVA (one test per marker with Tukey's multiple comparison correction).

We next evaluated whether the process of Treg engineering impacted on their ability to expand or changed their phenotypes. Transduced and untransduced Tregs from the same batches were expanded in parallel and their phenotypes compared. We found no significant differences between different Treg types, neither in their expansion properties (Fig. **2A**) nor in their phenotypes (Fig. 2B; Supporting Information Fig. S3). Notably, the expression of homing molecules relevant for the migration and function of CD4^+^CD25^+^CD127^lo^ Tregs including CD62L, CCR4, CCR9, CCR10, CLA, and β7 also remained unaffected by engineering with IL‐10 in the presence or absence of the A2‐CAR (Fig. 2C).

**Figure 3:**
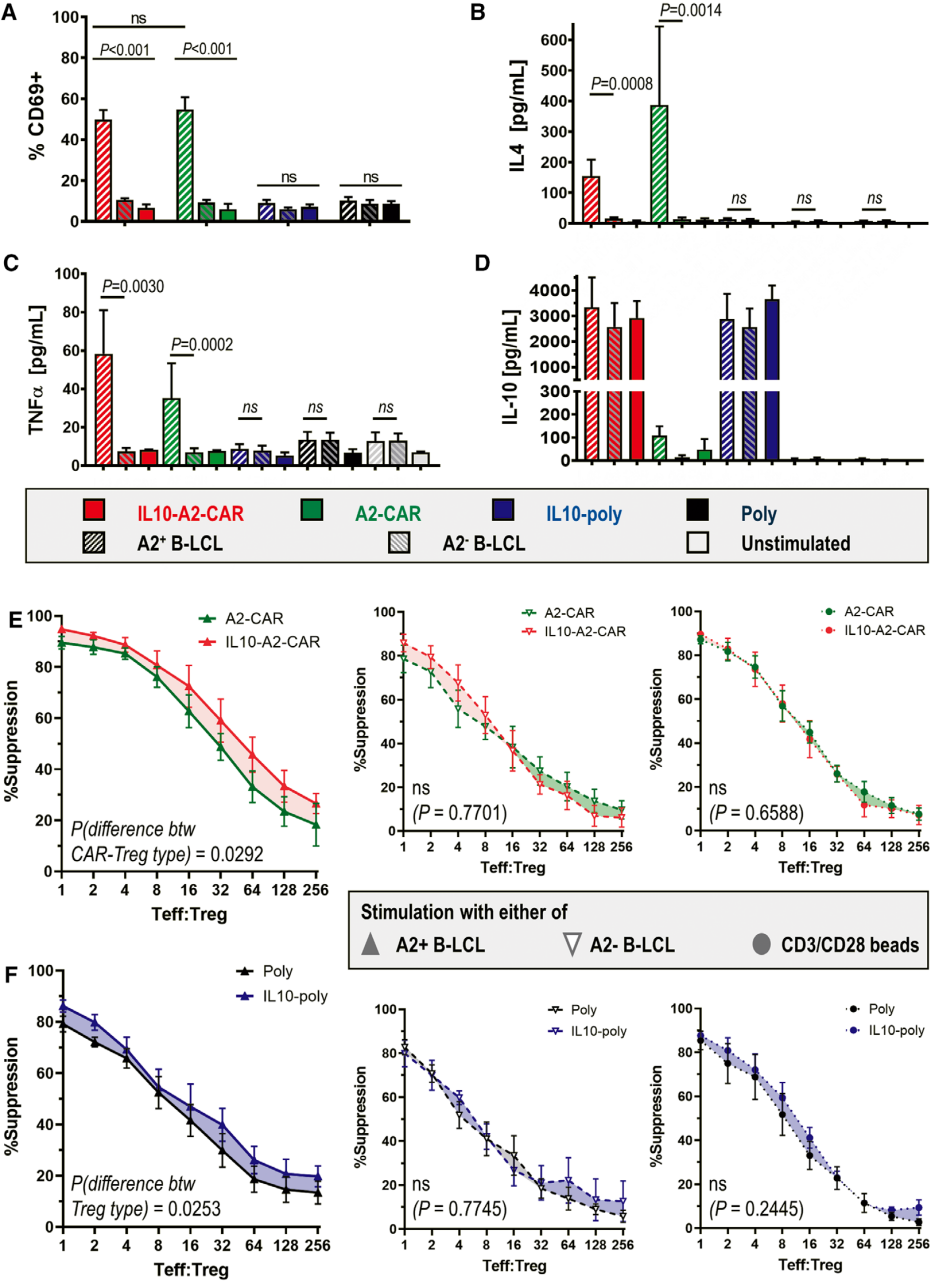
**Activation and suppression capacity of differently transduced Tregs. (A) **Transduced Tregs were cultured with indicated B‐LCL for 18 h before being harvested and stained for anti‐CD69. Cumulative data are shown from *n* = 8 different Treg batches (donors; one donor per experiment). Representative flow cytometry plots are shown in Supporting Information Fig. S4. **(B–D) **From experiments in (A), culture supernatants were collected and indicated cytokines were analyzed. Error bars represent SEM, *n* = 6 donors (one donor per experiment). Relative differences between treatments with A2^+^ or A2^−^ B‐LCL were analyzed by ratio paired *t*‐test for each Treg type. **(E and F)** Indicated Treg types with or without IL‐10 co‐expression were compared. Cells were co‐cultured with dye‐labeled Teffs and indicated B‐LCL for 5 days at indicated Teff:Treg ratios (cf. Materials and Methods section). Suppression of Teff proliferation was measured by Teff label dilution using flow cytometry. Shown is the suppression of Teff proliferation by Tregs stimulated with either A2^+^ or A2^− ^B‐LCL. Large panels (left column, large) show the comparison of IL‐10‐expressing Tregs and corresponding Tregs without IL‐10 co‐expression in the presence of A2^+ ^B‐LCLs. The other panels are controls whereby (middle column) show the same comparison in the presence of A2^− ^B‐LCLs and (right column) when B‐LCLs were replaced by CD3/CD28 activation beads. Data are from *n* = 6 different Treg batches (donors; one donor per experiment). Statistical analysis was performed by mixed‐model two‐way ANOVA with matched pairs per donor batch with *p*‐values added to figure panels.

Next, we assessed whether IL‐10 co‐expression would impact Treg activation. We co‐cultured each transduced Treg population with one of two irradiated B‐lymphoblastic cell lines (B‐LCLs) that expressed either HLA‐A2^+^ or HLA‐A2^−^ together with the same HLA‐DR haplotype (DR11), and subsequently analyzed CD69 upregulation in the Tregs as a measure of activation. As expected, both IL10‐A2‐CAR‐Tregs and A2‐CAR‐Tregs upregulated CD69 after co‐culture with the A2^+ ^B‐LCLs (49.9 ± 4.6% and 54.9 ± 5.9%, respectively) but not after co‐culture with the A2^− ^B‐LCLs (Fig. 3A, Supporting Information Fig. S4). Importantly, Tregs lacking the A2‐CAR did not show significant CD69 upregulation after co‐culture with A2^+ ^B‐LCLs. Moreover, A2‐CAR‐expressing Tregs produced IL‐4 and TNF‐α when co‐cultured with A2^+^ but not A2^− ^B‐LCLs, whereas Tregs lacking the A2‐CAR did not produce these cytokines when stimulated in this manner (Fig. 3B and C). Co‐expression of IL‐10 did not impact on IL‐4 or TNF‐α production. Both were higher in A2‐CAR‐Tregs than in Tregs without the CAR; however, this phenomenon did not appear to be detrimental to Treg function, while its significance is yet to be determined [23]. Due to the constitutive expression of the IL‐10 payload, secreted IL‐10 levels were comparable in all conditions between the IL‐10 co‐expressing Treg types. Notably, A2‐CAR Tregs also produced IL‐10 upon stimulation with A2^+^ B‐LCLs, although this was at much lower levels compared to IL‐10‐expressing Treg types (Fig. 3D). The various engineered Tregs produced very little IL‐17A on activation with either A2^+^ or A2^−^ B‐LCLs with no significant differences between conditions, and generally at levels very similar to untransduced Tregs (Supporting Information Fig. S5A). Notably, at day 20 only a small percentage of our engineered Tregs express PD‐1 (Supporting Information Fig. S5B), which could have been indicative of exhaustion if expressed in larger number of cells.

Taken together, these data demonstrate that the co‐expression of additional payloads such as IL‐10 and the imaging reporter did not impact on expansion capacity, Treg‐specific phenotype, and expected activation of the correspondingly engineered Tregs.

### IL‐10 expression enhances the suppressive capacity of A2‐CAR Tregs

To assess suppressive function, the various Treg types were co‐cultured with autologous pre‐labeled Teff cells and stimulated by either A2^+ ^B‐LCLs or A2^− ^B‐LCLs. The ability of Teffs to proliferate under these conditions was evident in all donors (Supporting Information Figs. S6 and S7). Despite the Treg cultures containing a small number of untransduced cells, both the Treg populations that expressed A2‐CARs suppressed Teffs significantly more effectively when cultured with A2^+ ^B‐LCLs than cultured with A2^−^ B‐LCLs (Supporting Information Fig. S8). Importantly, production of IL‐10 by Tregs significantly increased the suppressive capacity of A2‐CAR Tregs in the presence of A2^+ ^B‐LCLs (Fig. 3E [red>green] and F [blue>black]). Notably, the effect was not significant in the presence of A2^−^ B‐LCLs or upon stimulation with anti‐CD3/CD28 beads (Fig. 3E and F: bottom panels). The effect seen with the A2^−^ BLCLs was likely due to stimulation via HLA‐DR. Our findings were further supported by blocking the IL‐10 receptor (IL‐10R) through addition of a corresponding blocking antibody to the suppression assays (Supporting Information Fig. S9). In the presence of anti‐IL‐10R, the suppressive capacity of IL10‐A2‐CAR Tregs returned to levels similar to A2‐CAR Tregs, demonstrating specificity of the IL‐10‐dependent enhancement in suppressive capacity.

## Concluding remarks

Our in vitro data demonstrate that it is feasible to engineer Tregs efficiently with large expression cassettes using lentiviral technology (our largest provirus length was 8.67 kb) despite approaching provirus lengths empirically associated with inefficient virus production [37, 38]. We exploited this to transfer up to three different constructs as one ORF (CAR, cytokine, reporter) separated by 2A self‐cleaving sequences [39] into primary human Tregs at high efficiency and with all components being functional (**Fig**. 1). Tregs remained unchanged in expansion characteristics and overall phenotype (**Fig**. 2). We further demonstrated that the co‐expression of IL‐10 as an additional payload enhanced human Treg suppressive capacity (**Fig**. 3, Supporting Information Fig. S9). This was also evident in A2‐CAR‐Tregs, which were already significantly more suppressive than polyclonal Tregs if stimulated via their CARs by A2^+ ^B‐LCLs. It is noteworthy that suppression of A2^+^ B‐LCLs was also somewhat higher than suppression with A2^−^ B‐LCLs in Treg types without CAR expression, and we believe this is caused by a slightly stronger capacity of the latter B‐LCLs to activate T cells (Supporting Information Fig. S6B: middle panels). Importantly, when the CAR was present, the difference between the curves was much larger than the differences observed between IL‐10‐poly and Poly Treg lines.

In the future, we envisage CAR Tregs to be produced with precision using a specific antibody‐binding domain against a mismatched HLA haplotype of interest. CAR constructs that contain the most common MHC class I antigen‐binding domains may be highly beneficial to ensure full population coverage. In this context, it will be important to explore how activation of CAR‐Tregs through their endogenous TCR impacts on their function and efficacy, and whether TCR deletion needs to be considered (its feasibility has already been shown [40, 41]). A likely benefit of the TCR‐independent allospecificity mediated through CAR technology is the requirement for a reduced number of cells to be administrated. Nevertheless, it is paramount to engineer these Tregs for optimal efficacy. The way we have approached this, by co‐expression of IL‐10, is another step toward optimized CAR‐Treg production.

## Materials and methods

### Constructs

“IL10‐poly” (Fig. 1A: blue) was constructed by first amplifying human IL‐10 from pCAG.IL‐10.eGFP (gifted by Dr. K. Milward, vector map shown in Supporting Information Fig. S10) with flanking *SalI‐XhoI/SacII‐EcoRI* restriction sites (STEP‐2F and CTRL‐10R primers), and then subcloning this DNA (using *XhoI/SacII* sites) upstream to NIS‐TRFP into the pcDNA3.1(‐)MycHis‐based expression vector already containing NIS‐TRFP (previously reported as “intermediate construct” (IC) in [36]). For “A2‐CAR” (Fig. 1A: green), the CAR (without EGFP) was amplified from pLNT/SFFV.HLA‐A2‐CAR‐EGFP [24] with flanking *SalI‐XhoI/SacII‐EcoRI* restriction sites (CTRL‐A2F and STEP‐1R primers) and subcloned into pUC19 (”pUC19‐Intermediate”). From there, the cassette was subcloned using *XhoI/SacII* sites into IC upstream to NIS‐TRFP as for IL‐10 above. For “IL10‐A2‐CAR” (Fig. 1A: red), IL‐10 was amplified from pCAG.IL‐10.eGFP with flanking *SalI‐XhoI/BamHI* restriction sites (STEP‐2F and STEP‐2R) and subcloned into the pUC19‐intermediate, and the CAR was amplified from pLNT/SFFV.HLA‐A2‐CAR‐EGFP with flanking *BamHI/SacII‐EcoRI* restriction sites (STEP‐1F and STEP‐1R). The whole IL10‐A2‐CAR cassette was then subcloned into IC using *XhoI/SacII* sites, again upstream of NIS‐TRFP. Finally, all constructs were cut from their assembly vectors, gel purified, and subcloned into the lentiviral pLNT/SFFV backbone using *XhoI/NdeI*. In all these constructs, 2A technology was used to generate separate proteins from the same transcript [39]. Therefore, we chose 2A from *Thosea asigna* as it offers efficient construct separation and has been reported to elicit minimal immunogenicity [42]. All used primers are listed in Supporting Information Table S1. All constructs were confirmed by Sanger sequencing (Genewiz, UK) and their sequences are listed in Supporting Information Tables S2–S5.

### CD4^+^CD25^+^Treg and CD4^+^CD25^−^Teff isolation and culture

Cells were isolated from leukocyte‐enriched leukophoresis blood cones (National Blood Service, NHS Blood and Transplantation, Tooting, London, UK) with ethical approval (reference 9/H0707/86). CD4^+^T cells were isolated from total peripheral blood monocytes (PBMCs) by negative selection using Rosette‐Sep^TM^ (Rosette‐Sep^TM^, StemCell Technologies, UK) and CD4^+^T cells were enriched for CD25^+^T cells using CD25 Microbeads II (Miltenyi Biotec, UK). Collected CD4^+^CD25^−^T cells were preserved by freezing in liquid nitrogen until use. CD4^+^CD25^+^Tregs were cultured at 1 × 10^6^/mL in X‐VIVO^TM^ 15 (Lonza, UK) and activated with anti‐CD3/CD28 Dynabeads (1:1 bead/cell ratio; ThermoFisher, UK). Growth media were supplemented with 1000 U/mL IL‐2 (R&D Systems, Minnesota, USA) and 100 nM rapamycin (LC‐Laboratories, MA, USA) and replaced every 2 days. On days of harvest, Dynabeads were removed by magnetic force before any subsequent analyses.

### CAR‐Treg generation

Lentiviral particles were produced as previously described [43]. Virus titers were determined and Tregs transduced with the same multiplicity of infection (MOI) for all constructs. After 3 days of activation using anti‐CD3/CD28 Dynabeads (1:1 bead/cell ratio), Tregs were transduced in retronectin‐coated plates (50μg/μL; TakaraBio, France) with optimized viral titers. Transduced Tregs were cultured for an additional 7 days and then sorted on a FACSAriaIII (BD Biosciences, UK) using NIS‐TRFP reporter fluorescence for positive selection.

### Flow cytometry

Where possible, flow cytometry was performed according to published guidelines [44]. Cells were fixed and permeabilized using the Fix/Perm kit (eBioscience, UK) and subsequently stained in phosphate‐buffered saline (PBS; ThermoFisher) using the following fluorescently conjugated antibodies at concentrations ranging from 1 to 5 μg/mL: CD4‐DAPI (clone SK3), CD25‐PE‐CF594 (M‐A251), CD127‐BV786 (HIL‐7R‐M21), CTLA‐4‐BV421 (BNI3), CD45RA‐BUV737 (HI100), CD62L‐BV605 (DREG‐56), integrin β7‐BV650 (FIB504), CD39‐PE‐Cy7 (eBioA1) (eBioscience, UK), FOXP3‐APC (PCH101) (ThermoFisher); CLA‐PE‐Cy7(HECA‐452), CCR9‐PerCP‐Cy5.5 (L053E8), CCR10‐APC (6588‐5), and CD69‐PerCP (FN50); CD366‐APC (Tim‐3, clone F38‐2E2) and CD279‐APC/Cy7 (PD‐1, clone EH12.2H7) (BioLegend, UK); and CCR4‐Alexafluor700 (FAB1567N) (R&D Systems, UK). Dead cells were detected with Fixable Viability Stain 780 (BD Biosciences). To quantify the CAR expression, Tregs were labeled with an allophycocyanin (APC)‐conjugated dextramer specific for HLA‐A*0201/CINGVCWTV; APC‐conjugated HLA‐B*0702/APRGVRMAV served as a control (both dextramers from Immudex, Denmark). All samples were acquired on an LSRFortessa II (BD Biosciences) equipped with FACSDiva analysis software. Data were analyzed by FlowJo v9.7.5 (Tree Star, OR, USA).

Immunoblotting using Treg lysates (3 × 10^6^ cells) subjected to SDS‐PAGE gel electrophoresis and electrotransfer onto PVDF membranes followed by protein detection using a polyclonal rabbit anti‐human NIS (Imanis, MN, USA; #SJ1, 1 μg/mL) and monocloncal mouse anti‐GAPDH (Genetex, Taiwan ROC; clone GT239, 0.5 μg/mL) antibody each was performed as previously described [36].

### Cytokine analysis

Concentrations of IL‐4, IL‐10, IL‐17A, and tumor necrosis factor α (TNF‐α) in culture supernatants were analyzed using a human Cytokine Bead Array Kit (BD Biosciences).

### EBV transformed B‐cell line culture

For Treg activation and suppression assays, two B‐LCLs, SPO (HLA‐A2^+^DR11^+^) and BM21 (HLA‐A2^‐^DR11^+^) (gift of Dr. M. Martinez‐Llordella/King's College London), were used. They were grown in RPMI‐1640 (Sigma Aldrich, UK) supplemented with 10% (v/v) heat‐inactivated fetal‐calf serum (BioSera, UK), 100 μg/mL streptomycin, 100 IU/mL penicillin, and 2 mM l‐glutamine (ThermoFisher) in a humidified atmosphere containing 5% (v/v) CO_2_ at 37°C.

### Treg activation assay

SPO (HLA‐A2^+^DR11^+^) and BM21 (HLA‐A2^−^DR11^+^) cells were irradiated at 12,000 cGy for 45 min (Cell Rad, UK). Subsequently, Tregs or CAR‐Tregs were co‐cultured with one of the B‐cell types (1:4 Treg:B‐cells ratio) for 18 h in X‐VIVO^TM^ containing 5% (v/v) human AB serum only (rapamycin, IL‐2, and beads removed during prior harvest). Cells were harvested and stained with an antibody specific for CD69 (BioLegend, UK) before analysis using an LSR Fortessa II (BD Biosciences).

### Suppression assay

Suppressive capacity of Tregs or CAR‐Tregs was assessed by co‐culturing autologous Teffs with different amounts of Tregs or CAR‐Tregs (ratios ranging from 1:1 to 256:1). Teffs were pre‐labeled with CellTrace Violet (CTV; ThermoFisher). Both Teffs and Tregs were stimulated with either one of the irradiated B‐cell lines SPO (HLA‐A2^+^DR11^+^) or BM21 (HLA‐A2^−^DR11^+^) (Teffs:B‐cell ratios at 1:3), or anti‐CD3/CD28 beads (Teff:bead ratio at 40:1). Teff proliferation was measured through CTV label dilution by flow cytometry after 5 days as previously described [24]. In brief, Treg suppression was determined as a percentage defined by inverse of Teff proliferation as identified when gating for CTV, relative to stimulated Teffs alone. For the IL‐10 receptor blocking experiments, we used the monoclonal rat anti‐IL10R (CD210) IgG2_a,κ_ antibody clone 3F9 (BioLegend, UK) at 15 μg/mL final assay concentration.

### Radiotracer uptake assay

Note that 3 × 10^6^ indicated CAR T cells were transferred into Eppendorf tubes, washed with ice‐cold HBSS (ThermoFisher), and resuspended in 1 mL growth medium. Also 50 kBq generator‐eluted [^99m^Tc]TcO_4_^−^ (supplied by local King's Health Partners’ Radiopharmacy and used within two half‐lives) were added and cells incubated for 30 min at 37°C. Subsequently, cells were pelleted, supernatants collected, and cells washed twice with 1 mL HBSS before being resuspended in growth medium for γ‐counting (1282 Compugamma, LKB‐Wallac). Radiotracer uptake was calculated according to the following equation, in which Cpm represents decay‐corrected radioactivity counts per minute:

(1) $$\begin{array}{ccc} \%\mathrm{Tracer}\;\mathrm{uptake} & = & \frac{\mathrm{Cpm}\;\left[\mathrm{Cells}\right]}{\mathrm{Cpm}\;\left[\mathrm{Cells}\right]+\mathrm{Cpm}\;\left[\mathrm{Supernatant}\right]}\cdot 100\\ & & \;+\;\mathrm{Cpm}\left[\mathrm{Wash}1\right]+\mathrm{Cpm}\left[\mathrm{Wash}2\right] \end{array}$$

Statistical analyses were performed using Prism v8 software (GraphPad, CA, USA) with details added to figure legends and in the text.

## Conflict of Interest

GL and RIL are stakeholders of Quell Tx Ltd while YRM and SLT have been employed by Quell Tx Ltd after completing practical work leading to this manuscript. The other authors declare no commercial or financial conflict of interest.

## Author contributions

Conceptualization: GL and GOF. Data curation: AS, GL, GOF, and YRM. Formal analysis: AS, FI, GOF, and YRM. Funding acquisition: GL and GOF. Investigation: AS, CD, CL, SLT, and YRM. Methodology: CS, GOF, and QP. Project administration: GL and GOF. Supervision: GL and GOF. Validation: AS, FI, GL, GOF, and YRM. Visualization: GOF and YRM. Writing‐original draft: FI, GL, GOF, and YRM. Writing–review and editing: all authors.

### Peer review

The peer review history for this article is available at https://publons.com/publon/10.1002/eji.202048934

**Abbreviations**
B‐LCL: B‐lymphoblastic cell line
CAR: chimeric antigen receptor
CTV: CellTrace Violet

## Supporting information



Supporting Information

## Data Availability

The data that support the findings of this study are available from the corresponding author upon reasonable request.
