# Supplementary material for: Chimeric antigen receptor‐modified human regulatory T cells that constitutively express IL‐10 maintain their phenotype and are potently suppressive
Source: Eur J Immunol. 2021 Aug 8;51(10):2522–30. doi: 10.1002/eji.202048934 (PMC8581768; doi:10.1002/eji.202048934)
Supplement: Supplementary file 1 — Supporting Information [file EJI-51-2522-s001.docx]

**SUPPORTING INFORMATION**

to the manuscript

**“Chimeric antigen receptor-modified human regulatory T cells that constitutively express interleukin-10 maintain their phenotype and are potently suppressive.”**

by

Yasmin R. Mohseni, Adeel Saleem, Sim L. Tung, Caroline Dudreiulh, Cameron Lang, Qi Peng, Alessia Volpe, George Adigbli, Amy Cross, Joanna Hester, Farzin Farzaneh, Cristiano Scotta, Robert I. Lechler, Fadi Issa^†^, Gilbert O. Fruhwirth^†^, Giovanna Lombardi^†^.

This supplement contains ten additional figures and five additional tables supporting the data in the main manuscript.

**Supplementary Tab.S1.** Sequences of primers used to generate the constructs “A2-CAR”, “IL10-poly” and “IL10-A2-CAR” as illustrated in Fig.1A.

| **Name** | **Coverage** | **Type** | **Length/bp** | **Sequence (5’-3’)** |
| --- | --- | --- | --- | --- |
| “STEP-2F” | *SalI-XhoI*-IL-10 | FWD | 46 | GTACGACGCG TCGACCTCGA GACCGCCATG CACAGCTCAG CACTGC |
| “CTRL-10R” | IL-10-*SacII-EcoRI* | REV | 52 | GCGCCGGAAT TCCCGCGGGC TGCGCTTTCT CCGGTTTCGT ATCTTCATTG TC |
| “CTRL-A2F” | *SalI-XhoI*-CAR | FWD | 46 | GTACGACGCG TCGACCTCGA GACCGCCATG GCCCTGCCCG TGACCG |
| “STEP-1R” | CAR-*SacII-EcoRI* | REV | 37 | GCGCCGGAAT TCCCGCGGGC GAGGGGGCAG GGCCTGC |
| “STEP-1F” | *BamHI*-T2A-CAR | FWD | 88 | GCGCGCGGAT CCGGCGAGGG CCGGGGCAGC CTGCTGACCT GCGGCGACGT GGAAGAGAAC CCCGGACCCA TGGCCCTGCC CGTGACCG |
| “STEP-2R” | IL-10-*BamHI* | REV | 46 | CGCGCGGGAT CCGCTGCGCT TTCTCCGGTT TCGTATCTTG ATTGTC |

**Supplementary Tab.S2.** Sequence of the “A2-CAR” construct with colours matching Fig.1A and relevant linkers underlined (green/bold: HLA-A2 CAR, black: NIS-TagRFP fusion reporter wherein NIS is bold).

**ATGGCCCTGCCCGTGACCGCCCTGCTGCTGCCCCTGGCCCTGCTGCTGCACGCCGCCCGGCCTCAGGTGCAGCTGGTGCAGAGCGGCGGCGGCGTGGTGCAGCCCGGAGGCAGCCTGAGGGTGAGCTGCGCCGCCAGCGGCGTGACCCTGAGCGACTACGGCATGCACTGGGTGCGGCAGGCTCCCGGCAAGGGCCTGGAGTGGATGGCCTTCATCCGGAACGACGGCAGCGACAAGTACTACGCCGACAGCGTGAAGGGCCGGTTCACCATCAGCCGGGACAACAGCAAGAAGACCGTGAGCCTGCAGATGAGCAGCCTGCGGGCTGAGGACACCGCCGTGTACTACTGCGCCAAGAACGGCGAGAGCGGCCCTCTGGACTACTGGTACTTCGACCTGTGGGGCAGGGGAACCCTGGTGACCGTGAGCAGCGGCGGCGGAGGCAGCGGTGGCGGAGGCAGCGGCGGAGGCGGTAGCGACGTGGTGATGACCCAGAGCCCCAGCAGCCTGAGCGCCAGCGTGGGCGACCGGGTGACCATCACCTGCCAGGCCAGCCAGGACATCAGCAACTACCTGAACTGGTACCAGCAGAAGCCCGGCAAGGCCCCTAAGCTGCTGATCTACGACGCCAGCAACCTGGAGACCGGCGTGCCAAGCCGGTTCAGCGGCAGCGGAAGCGGCACCGACTTCACCTTCACCATCAGCAGCCTGCAGCCTGAGGACATCGCCACCTACTACTGCCAGCAGTACGACAACCTGCCTCCCACCTTCGGCGGAGGCACCAAGCTGACCGTGCTGGGCGCGGCCGCCATCGAGGTGGAGCAGAAGCTGATCAGCGAGGAGGACCTGCTGGACAACGAGAAGAGCAACGGCACCATCATCCACGTGAAGGGCAAGCACCTGTGCCCCAGCCCCCTGTTCCCCGGCCCCAGCAAGCCCTTCTGGGTGCTGGTGGTGGTGGGCGGCGTGCTGGCCTGCTACAGCCTGCTGGTGACCGTGGCCTTCATCATCTTCTGGGTGCGGAGCAAGCGGAGCCGGCTGCTGCACAGCGACTACATGAACATGACCCCCCGGCGGCCTGGGCCCACCCGCAAGCATTACCAGCCCTATGCCCCACCACGCGACTTCGCAGCCTATCGCTCCAGAGTGAAGTTCAGCAGGAGCGCAGAGCCCCCCGCGTACCAGCAGGGCCAGAACCAGCTCTATAACGAGCTCAATCTAGGACGAAGAGAGGAGTACGATGTTTTGGACAAGAGACGTGGCCGGGACCCTGAGATGGGGGGAAAGCCGAGAAGGAAGAACCCTCAGGAAGGCCTGTACAATGAACTGCAGAAAGATAAGATGGCGGAGGCCTACAGTGAGATTGGGATGAAAGGCGAGCGCCGGAGGGGCAAGGGGCACGATGGCCTTTACCAGGGTCTCAGTACAGCCACCAAGGACACCTACGACGCCCTTCACATGCAGGCCCTGCCCCCTCGC**CCGCGGGAGGGCCGGGGCAGCCTGCTGACCTGCGGCGACGTGGAAGAGAACCCCGGACCC**ATGGAGGCCGTGGAGACCGGGGAACGGCCCACCTTCGGAGCCTGGGACTACGGGGTCTTTGCCCTCATGCTCCTGGTGTCCACTGGCATCGGGCTGTGGGTCGGGCTGGCTCGGGGCGGGCAGCGCAGCGCTGAGGACTTCTTCACCGGGGGCCGGCGCCTGGCGGCCCTGCCCGTGGGCCTGTCGCTGTCTGCCAGCTTCATGTCGGCCGTGCAGGTGCTGGGCGTGCCGTCGGAGGCCTATCGCTATGGCCTCAAGTTCCTCTGGATGTGCCTGGGCCAGCTTCTGAACTCGGTCCTCACCGCCCTGCTCTTCATGCCCGTCTTCTACCGCCTGGGCCTCACCAGCACCTACGAGTACCTGGAGATGCGCTTCAGCCGCGCAGTGCGGCTCTGCGGGACTTTGCAGTACATTGTAGCCACGATGCTGTACACCGGCATCGTAATCTACGCACCGGCCCTCATCCTGAACCAAGTGACCGGGCTGGACATCTGGGCGTCGCTCCTGTCCACCGGAATTATCTGCACCTTCTACACGGCTGTGGGCGGCATGAAGGCTGTGGTCTGGACTGATGTGTTCCAGGTCGTGGTGATGCTAAGTGGCTTCTGGGTTGTCCTGGCACGCGGTGTCATGCTTGTGGGCGGGCCCCGCCAGGTGCTCACGCTGGCCCAGAACCACTCCCGGATCAACCTCATGGACTTTAACCCTGACCCGAGGAGCCGCTATACATTCTGGACTTTTGTGGTGGGTGGCACGTTGGTGTGGCTCTCCATGTATGGCGTGAACCAGGCGCAGGTGCAGCGCTACGTGGCTTGCCGCACAGAGAAGCAGGCCAAGCTGGCCCTGCTCATCAACCAGGTCGGCCTGTTCCTGATCGTGTCCAGCGCTGCCTGCTGTGGCATCGTCATGTTTGTGTTCTACACTGACTGCGACCCTCTCCTCCTGGGGCGCATCTCTGCCCCAGACCAGTACATGCCTCTGCTGGTGCTGGACATCTTCGAAGATCTGCCTGGAGTCCCCGGGCTTTTCCTGGCCTGTGCTTACAGTGGCACCCTCAGCACAGCATCCACCAGCATCAATGCTATGGCTGCAGTCACTGTAGAAGACCTCATCAAACCTCGGCTGCGGAGCCTGGCACCCAGGAAACTCGTGATTATCTCCAAGGGGCTCTCACTCATCTACGGATCGGCCTGTCTCACCGTGGCAGCCCTGTCCTCACTGCTCGGAGGAGGTGTCCTTCAGGGCTCCTTCACCGTCATGGGAGTCATCAGCGGCCCCCTGCTGGGAGCCTTCATCTTGGGAATGTTCCTGCCGGCCTGCAACACACCGGGCGTCCTCGCGGGACTAGGCGCGGGCTTGGCGCTGTCGCTGTGGGTGGCCTTGGGCGCCACGCTGTACCCACCCAGCGAGCAGACCATGAGGGTCCTGCCATCGTCGGCTGCCCGCTGCGTGGCTCTCTCAGTCAACGCCTCTGGCCTCCTGGACCCGGCTCTCCTCCCTGCTAACGACTCCAGCAGGGCCCCCAGCTCAGGAATGGACGCCAGCCGACCCGCCTTAGCTGACAGCTTCTATGCCATCTCCTATCTCTATTACGGTGCCCTGGGCACGCTGACCACTGTGCTGTGCGGAGCCCTCATCAGCTGCCTGACAGGCCCCACCAAGCGCAGCACCCTGGCCCCGGGATTGTTGTGGTGGGACCTCGCACGGCAGACAGCATCAGTGGCCCCCAAGGAAGAAGTGGCCATCCTGGATGACAACTTGGTCAAGGGTCCTGAAGAACTCCCCACTGGAAACAAGAAGCCCCCTGGCTTCCTGCCCACCAATGAGGATCGTCTGTTTTTCTTGGGGCAGAAGGAGCTGGAGGGGGCTGGCTCTTGGACCCCTTGTGTTGGACATGATGGTGGTCGAGACCAGCAGGAGACAAACCTC**GGCATTCTGCAGTCcACGGTtCCGCGcGCCCGGGATCCACCGGTCGCCACCATGGTGTCTAAGGGCGAAGAGCTGATTAAGGAGAACATGCACATGAAGCTGTACATGGAGGGCACCGTGAACAACCACCACTTCAAGTGCACATCCGAGGGCGAAGGCAAGCCCTACGAGGGCACCCAGACCATGAGAATCAAGGTGGTCGAGGGCGGCCCTCTCCCCTTCGCCTTCGACATCCTGGCTACCAGCTTCATGTACGGCAGCAGAACCTTCATCAACCACACCCAGGGCATCCCCGACTTCTTTAAGCAGTCCTTCCCTGAGGGCTTCACATGGGAGAGAGTCACCACATACGAAGACGGGGGCGTGCTGACCGCTACCCAGGACACCAGCCTCCAGGACGGCTGCCTCATCTACAACGTCAAGATCAGAGGGGTGAACTTCCCATCCAACGGCCCTGTGATGCAGAAGAAAACACTCGGCTGGGAGGCCAACACCGAGATGCTGTACCCCGCTGACGGCGGCCTGGAAGGCAGAAGCGACATGGCCCTGAAGCTCGTGGGCGGGGGCCACCTGATCTGCAACTTCAAGACCACATACAGATCCAAGAAACCCGCTAAGAACCTCAAGATGCCCGGCGTCTACTATGTGGACCACAGACTGGAAAGAATCAAGGAGGCCGACAAAGAGACCTACGTCGAGCAGCACGAGGTGGCTGTGGCCAGATACTGCGACCTCCCTAGCAAACTGGGGCACAAACTTAATTGA.

**Supplementary Tab.S3.** Sequence of “IL10-poly” construct with colours matching Fig.1A and relevant linkers underlined (blue/bold: IL10, black: NIS-TagRFP fusion reporter wherein NIS is bold).

**ATGCACAGCTCAGCACTGCTCTGTTGCCTGGTCCTCCTGACTGGGGTGAGGGCCAGCCCAGGCCAGGGCACCCAGTCTGAGAACAGCTGCACCCACTTCCCAGGCAACCTGCCTAACATGCTTCGAGATCTCCGAGATGCCTTCAGCAGAGTGAAGACTTTCTTTCAAATGAAGGATCAGCTGGACAACTTGTTGTTAAAGGAGTCCTTGCTGGAGGACTTTAAGGGTTACCTGGGTTGCCAAGCCTTGTCTGAGATGATCCAGTTTTACCTGGAGGAGGTGATGCCCCAAGCTGAGAACCAAGACCCAGACATCAAGGCGCATGTGAACTCCCTGGGGGAGAACCTGAAGACCCTCAGGCTGAGGCTACGGCGCTGTCATCGATTTCTTCCCTGTGAAAACAAGAGCAAGGCCGTGGAGCAGGTGAAGAATGCCTTTAATAAGCTCCAAGAGAAAGGCATCTACAAAGCCATGAGTGAGTTTGACATCTTCATCAACTACATAGAAGCCTACATGACAATGAAGATACGAAAC**CGGAGAAAGCGCAGCGGATCCGGCccgcggGAGGGCCGGGGCAGCCTGCTGACCTGCGGCGACGTGGAAGAGAACCCCGGACCC**ATGGAGGCCGTGGAGACCGGGGAACGGCCCACCTTCGGAGCCTGGGACTACGGGGTCTTTGCCCTCATGCTCCTGGTGTCCACTGGCATCGGGCTGTGGGTCGGGCTGGCTCGGGGCGGGCAGCGCAGCGCTGAGGACTTCTTCACCGGGGGCCGGCGCCTGGCGGCCCTGCCCGTGGGCCTGTCGCTGTCTGCCAGCTTCATGTCGGCCGTGCAGGTGCTGGGCGTGCCGTCGGAGGCCTATCGCTATGGCCTCAAGTTCCTCTGGATGTGCCTGGGCCAGCTTCTGAACTCGGTCCTCACCGCCCTGCTCTTCATGCCCGTCTTCTACCGCCTGGGCCTCACCAGCACCTACGAGTACCTGGAGATGCGCTTCAGCCGCGCAGTGCGGCTCTGCGGGACTTTGCAGTACATTGTAGCCACGATGCTGTACACCGGCATCGTAATCTACGCACCGGCCCTCATCCTGAACCAAGTGACCGGGCTGGACATCTGGGCGTCGCTCCTGTCCACCGGAATTATCTGCACCTTCTACACGGCTGTGGGCGGCATGAAGGCTGTGGTCTGGACTGATGTGTTCCAGGTCGTGGTGATGCTAAGTGGCTTCTGGGTTGTCCTGGCACGCGGTGTCATGCTTGTGGGCGGGCCCCGCCAGGTGCTCACGCTGGCCCAGAACCACTCCCGGATCAACCTCATGGACTTTAACCCTGACCCGAGGAGCCGCTATACATTCTGGACTTTTGTGGTGGGTGGCACGTTGGTGTGGCTCTCCATGTATGGCGTGAACCAGGCGCAGGTGCAGCGCTACGTGGCTTGCCGCACAGAGAAGCAGGCCAAGCTGGCCCTGCTCATCAACCAGGTCGGCCTGTTCCTGATCGTGTCCAGCGCTGCCTGCTGTGGCATCGTCATGTTTGTGTTCTACACTGACTGCGACCCTCTCCTCCTGGGGCGCATCTCTGCCCCAGACCAGTACATGCCTCTGCTGGTGCTGGACATCTTCGAAGATCTGCCTGGAGTCCCCGGGCTTTTCCTGGCCTGTGCTTACAGTGGCACCCTCAGCACAGCATCCACCAGCATCAATGCTATGGCTGCAGTCACTGTAGAAGACCTCATCAAACCTCGGCTGCGGAGCCTGGCACCCAGGAAACTCGTGATTATCTCCAAGGGGCTCTCACTCATCTACGGATCGGCCTGTCTCACCGTGGCAGCCCTGTCCTCACTGCTCGGAGGAGGTGTCCTTCAGGGCTCCTTCACCGTCATGGGAGTCATCAGCGGCCCCCTGCTGGGAGCCTTCATCTTGGGAATGTTCCTGCCGGCCTGCAACACACCGGGCGTCCTCGCGGGACTAGGCGCGGGCTTGGCGCTGTCGCTGTGGGTGGCCTTGGGCGCCACGCTGTACCCACCCAGCGAGCAGACCATGAGGGTCCTGCCATCGTCGGCTGCCCGCTGCGTGGCTCTCTCAGTCAACGCCTCTGGCCTCCTGGACCCGGCTCTCCTCCCTGCTAACGACTCCAGCAGGGCCCCCAGCTCAGGAATGGACGCCAGCCGACCCGCCTTAGCTGACAGCTTCTATGCCATCTCCTATCTCTATTACGGTGCCCTGGGCACGCTGACCACTGTGCTGTGCGGAGCCCTCATCAGCTGCCTGACAGGCCCCACCAAGCGCAGCACCCTGGCCCCGGGATTGTTGTGGTGGGACCTCGCACGGCAGACAGCATCAGTGGCCCCCAAGGAAGAAGTGGCCATCCTGGATGACAACTTGGTCAAGGGTCCTGAAGAACTCCCCACTGGAAACAAGAAGCCCCCTGGCTTCCTGCCCACCAATGAGGATCGTCTGTTTTTCTTGGGGCAGAAGGAGCTGGAGGGGGCTGGCTCTTGGACCCCTTGTGTTGGACATGATGGTGGTCGAGACCAGCAGGAGACAAACCTC**GGcATTCTGCAGTCcACGGTtCCGCGcGCCCGGGATCCACCGGTCGCCACCATGGTGTCTAAGGGCGAAGAGCTGATTAAGGAGAACATGCACATGAAGCTGTACATGGAGGGCACCGTGAACAACCACCACTTCAAGTGCACATCCGAGGGCGAAGGCAAGCCCTACGAGGGCACCCAGACCATGAGAATCAAGGTGGTCGAGGGCGGCCCTCTCCCCTTCGCCTTCGACATCCTGGCTACCAGCTTCATGTACGGCAGCAGAACCTTCATCAACCACACCCAGGGCATCCCCGACTTCTTTAAGCAGTCCTTCCCTGAGGGCTTCACATGGGAGAGAGTCACCACATACGAAGACGGGGGCGTGCTGACCGCTACCCAGGACACCAGCCTCCAGGACGGCTGCCTCATCTACAACGTCAAGATCAGAGGGGTGAACTTCCCATCCAACGGCCCTGTGATGCAGAAGAAAACACTCGGCTGGGAGGCCAACACCGAGATGCTGTACCCCGCTGACGGCGGCCTGGAAGGCAGAAGCGACATGGCCCTGAAGCTCGTGGGCGGGGGCCACCTGATCTGCAACTTCAAGACCACATACAGATCCAAGAAACCCGCTAAGAACCTCAAGATGCCCGGCGTCTACTATGTGGACCACAGACTGGAAAGAATCAAGGAGGCCGACAAAGAGACCTACGTCGAGCAGCACGAGGTGGCTGTGGCCAGATACTGCGACCTCCCTAGCAAACTGGGGCACAAACTTAATTGA.

**Supplementary Tab.S4.** Sequence of “IL10-A2-CAR” construct with colours matching Fig.1A and relevant linkers underlined (blue/bold: IL10, green/bold: HLA-A2 CAR, black: NIS-TagRFP fusion reporter wherein NIS is bold).

**ATGCACAGCTCAGCACTGCTCTGTTGCCTGGTCCTCCTGACTGGGGTGAGGGCCAGCCCAGGCCAGGGCACCCAGTCTGAGAACAGCTGCACCCACTTCCCAGGCAACCTGCCTAACATGCTTCGAGATCTCCGAGATGCCTTCAGCAGAGTGAAGACTTTCTTTCAAATGAAGGATCAGCTGGACAACTTGTTGTTAAAGGAGTCCTTGCTGGAGGACTTTAAGGGTTACCTGGGTTGCCAAGCCTTGTCTGAGATGATCCAGTTTTACCTGGAGGAGGTGATGCCCCAAGCTGAGAACCAAGACCCAGACATCAAGGCGCATGTGAACTCCCTGGGGGAGAACCTGAAGACCCTCAGGCTGAGGCTACGGCGCTGTCATCGATTTCTTCCCTGTGAAAACAAGAGCAAGGCCGTGGAGCAGGTGAAGAATGCCTTTAATAAGCTCCAAGAGAAAGGCATCTACAAAGCCATGAGTGAGTTTGACATCTTCATCAACTACATAGAAGCCTACATGACAATGAAGATACGAAAC**CGGAGAAAGCGCAGCGGATCCGGCGAGGGCCGGGGCAGCCTGCTGACCTGCGGCGACGTGGAAGAGAACCCCGGACCC**ATGGCCCTGCCCGTGACCGCCCTGCTGCTGCCCCTGGCCCTGCTGCTGCACGCCGCCCGGCCTCAGGTGCAGCTGGTGCAGAGCGGCGGCGGCGTGGTGCAGCCCGGAGGCAGCCTGAGGGTGAGCTGCGCCGCCAGCGGCGTGACCCTGAGCGACTACGGCATGCACTGGGTGCGGCAGGCTCCCGGCAAGGGCCTGGAGTGGATGGCCTTCATCCGGAACGACGGCAGCGACAAGTACTACGCCGACAGCGTGAAGGGCCGGTTCACCATCAGCCGGGACAACAGCAAGAAGACCGTGAGCCTGCAGATGAGCAGCCTGCGGGCTGAGGACACCGCCGTGTACTACTGCGCCAAGAACGGCGAGAGCGGCCCTCTGGACTACTGGTACTTCGACCTGTGGGGCAGGGGAACCCTGGTGACCGTGAGCAGCGGCGGCGGAGGCAGCGGTGGCGGAGGCAGCGGCGGAGGCGGTAGCGACGTGGTGATGACCCAGAGCCCCAGCAGCCTGAGCGCCAGCGTGGGCGACCGGGTGACCATCACCTGCCAGGCCAGCCAGGACATCAGCAACTACCTGAACTGGTACCAGCAGAAGCCCGGCAAGGCCCCTAAGCTGCTGATCTACGACGCCAGCAACCTGGAGACCGGCGTGCCAAGCCGGTTCAGCGGCAGCGGAAGCGGCACCGACTTCACCTTCACCATCAGCAGCCTGCAGCCTGAGGACATCGCCACCTACTACTGCCAGCAGTACGACAACCTGCCTCCCACCTTCGGCGGAGGCACCAAGCTGACCGTGCTGGGCGCGGCCGCCATCGAGGTGGAGCAGAAGCTGATCAGCGAGGAGGACCTGCTGGACAACGAGAAGAGCAACGGCACCATCATCCACGTGAAGGGCAAGCACCTGTGCCCCAGCCCCCTGTTCCCCGGCCCCAGCAAGCCCTTCTGGGTGCTGGTGGTGGTGGGCGGCGTGCTGGCCTGCTACAGCCTGCTGGTGACCGTGGCCTTCATCATCTTCTGGGTGCGGAGCAAGCGGAGCCGGCTGCTGCACAGCGACTACATGAACATGACCCCCCGGCGGCCTGGGCCCACCCGCAAGCATTACCAGCCCTATGCCCCACCACGCGACTTCGCAGCCTATCGCTCCAGAGTGAAGTTCAGCAGGAGCGCAGAGCCCCCCGCGTACCAGCAGGGCCAGAACCAGCTCTATAACGAGCTCAATCTAGGACGAAGAGAGGAGTACGATGTTTTGGACAAGAGACGTGGCCGGGACCCTGAGATGGGGGGAAAGCCGAGAAGGAAGAACCCTCAGGAAGGCCTGTACAATGAACTGCAGAAAGATAAGATGGCGGAGGCCTACAGTGAGATTGGGATGAAAGGCGAGCGCCGGAGGGGCAAGGGGCACGATGGCCTTTACCAGGGTCTCAGTACAGCCACCAAGGACACCTACGACGCCCTTCACATGCAGGCCCTGCCCCCTCGC**CCGCGGGAGGGCCGGGGCAGCCTGCTGACCTGCGGCGACGTGGAAGAGAACCCCGGACCC**ATGGAGGCCGTGGAGACCGGGGAACGGCCCACCTTCGGAGCCTGGGACTACGGGGTCTTTGCCCTCATGCTCCTGGTGTCCACTGGCATCGGGCTGTGGGTCGGGCTGGCTCGGGGCGGGCAGCGCAGCGCTGAGGACTTCTTCACCGGGGGCCGGCGCCTGGCGGCCCTGCCCGTGGGCCTGTCGCTGTCTGCCAGCTTCATGTCGGCCGTGCAGGTGCTGGGCGTGCCGTCGGAGGCCTATCGCTATGGCCTCAAGTTCCTCTGGATGTGCCTGGGCCAGCTTCTGAACTCGGTCCTCACCGCCCTGCTCTTCATGCCCGTCTTCTACCGCCTGGGCCTCACCAGCACCTACGAGTACCTGGAGATGCGCTTCAGCCGCGCAGTGCGGCTCTGCGGGACTTTGCAGTACATTGTAGCCACGATGCTGTACACCGGCATCGTAATCTACGCACCGGCCCTCATCCTGAACCAAGTGACCGGGCTGGACATCTGGGCGTCGCTCCTGTCCACCGGAATTATCTGCACCTTCTACACGGCTGTGGGCGGCATGAAGGCTGTGGTCTGGACTGATGTGTTCCAGGTCGTGGTGATGCTAAGTGGCTTCTGGGTTGTCCTGGCACGCGGTGTCATGCTTGTGGGCGGGCCCCGCCAGGTGCTCACGCTGGCCCAGAACCACTCCCGGATCAACCTCATGGACTTTAACCCTGACCCGAGGAGCCGCTATACATTCTGGACTTTTGTGGTGGGTGGCACGTTGGTGTGGCTCTCCATGTATGGCGTGAACCAGGCGCAGGTGCAGCGCTACGTGGCTTGCCGCACAGAGAAGCAGGCCAAGCTGGCCCTGCTCATCAACCAGGTCGGCCTGTTCCTGATCGTGTCCAGCGCTGCCTGCTGTGGCATCGTCATGTTTGTGTTCTACACTGACTGCGACCCTCTCCTCCTGGGGCGCATCTCTGCCCCAGACCAGTACATGCCTCTGCTGGTGCTGGACATCTTCGAAGATCTGCCTGGAGTCCCCGGGCTTTTCCTGGCCTGTGCTTACAGTGGCACCCTCAGCACAGCATCCACCAGCATCAATGCTATGGCTGCAGTCACTGTAGAAGACCTCATCAAACCTCGGCTGCGGAGCCTGGCACCCAGGAAACTCGTGATTATCTCCAAGGGGCTCTCACTCATCTACGGATCGGCCTGTCTCACCGTGGCAGCCCTGTCCTCACTGCTCGGAGGAGGTGTCCTTCAGGGCTCCTTCACCGTCATGGGAGTCATCAGCGGCCCCCTGCTGGGAGCCTTCATCTTGGGAATGTTCCTGCCGGCCTGCAACACACCGGGCGTCCTCGCGGGACTAGGCGCGGGCTTGGCGCTGTCGCTGTGGGTGGCCTTGGGCGCCACGCTGTACCCACCCAGCGAGCAGACCATGAGGGTCCTGCCATCGTCGGCTGCCCGCTGCGTGGCTCTCTCAGTCAACGCCTCTGGCCTCCTGGACCCGGCTCTCCTCCCTGCTAACGACTCCAGCAGGGCCCCCAGCTCAGGAATGGACGCCAGCCGACCCGCCTTAGCTGACAGCTTCTATGCCATCTCCTATCTCTATTACGGTGCCCTGGGCACGCTGACCACTGTGCTGTGCGGAGCCCTCATCAGCTGCCTGACAGGCCCCACCAAGCGCAGCACCCTGGCCCCGGGATTGTTGTGGTGGGACCTCGCACGGCAGACAGCATCAGTGGCCCCCAAGGAAGAAGTGGCCATCCTGGATGACAACTTGGTCAAGGGTCCTGAAGAACTCCCCACTGGAAACAAGAAGCCCCCTGGCTTCCTGCCCACCAATGAGGATCGTCTGTTTTTCTTGGGGCAGAAGGAGCTGGAGGGGGCTGGCTCTTGGACCCCTTGTGTTGGACATGATGGTGGTCGAGACCAGCAGGAGACAAACCTC**GGCATTCTGCAGTCCACGGTTCCGCGCGCCCGGGATCCACCGGTCGCCACCATGGTGTCTAAGGGCGAAGAGCTGATTAAGGAGAACATGCACATGAAGCTGTACATGGAGGGCACCGTGAACAACCACCACTTCAAGTGCACATCCGAGGGCGAAGGCAAGCCCTACGAGGGCACCCAGACCATGAGAATCAAGGTGGTCGAGGGCGGCCCTCTCCCCTTCGCCTTCGACATCCTGGCTACCAGCTTCATGTACGGCAGCAGAACCTTCATCAACCACACCCAGGGCATCCCCGACTTCTTTAAGCAGTCCTTCCCTGAGGGCTTCACATGGGAGAGAGTCACCACATACGAAGACGGGGGCGTGCTGACCGCTACCCAGGACACCAGCCTCCAGGACGGCTGCCTCATCTACAACGTCAAGATCAGAGGGGTGAACTTCCCATCCAACGGCCCTGTGATGCAGAAGAAAACACTCGGCTGGGAGGCCAACACCGAGATGCTGTACCCCGCTGACGGCGGCCTGGAAGGCAGAAGCGACATGGCCCTGAAGCTCGTGGGCGGGGGCCACCTGATCTGCAACTTCAAGACCACATACAGATCCAAGAAACCCGCTAAGAACCTCAAGATGCCCGGCGTCTACTATGTGGACCACAGACTGGAAAGAATCAAGGAGGCCGACAAAGAGACCTACGTCGAGCAGCACGAGGTGGCTGTGGCCAGATACTGCGACCTCCCTAGCAAACTGGGGCACAAACTTAATTGA.

**Supplementary Tab.S5.** Sequence of the NIS-TagRFP reporter only (named “Poly” in Fig.1A; NIS is bold, linker underlined).

**ATGGAGGCCGTGGAGACCGGGGAACGGCCCACCTTCGGAGCCTGGGACTACGGGGTCTTTGCCCTCATGCTCCTGGTGTCCACTGGCATCGGGCTGTGGGTCGGGCTGGCTCGGGGCGGGCAGCGCAGCGCTGAGGACTTCTTCACCGGGGGCCGGCGCCTGGCGGCCCTGCCCGTGGGCCTGTCGCTGTCTGCCAGCTTCATGTCGGCCGTGCAGGTGCTGGGCGTGCCGTCGGAGGCCTATCGCTATGGCCTCAAGTTCCTCTGGATGTGCCTGGGCCAGCTTCTGAACTCGGTCCTCACCGCCCTGCTCTTCATGCCCGTCTTCTACCGCCTGGGCCTCACCAGCACCTACGAGTACCTGGAGATGCGCTTCAGCCGCGCAGTGCGGCTCTGCGGGACTTTGCAGTACATTGTAGCCACGATGCTGTACACCGGCATCGTAATCTACGCACCGGCCCTCATCCTGAACCAAGTGACCGGGCTGGACATCTGGGCGTCGCTCCTGTCCACCGGAATTATCTGCACCTTCTACACGGCTGTGGGCGGCATGAAGGCTGTGGTCTGGACTGATGTGTTCCAGGTCGTGGTGATGCTAAGTGGCTTCTGGGTTGTCCTGGCACGCGGTGTCATGCTTGTGGGCGGGCCCCGCCAGGTGCTCACGCTGGCCCAGAACCACTCCCGGATCAACCTCATGGACTTTAACCCTGACCCGAGGAGCCGCTATACATTCTGGACTTTTGTGGTGGGTGGCACGTTGGTGTGGCTCTCCATGTATGGCGTGAACCAGGCGCAGGTGCAGCGCTACGTGGCTTGCCGCACAGAGAAGCAGGCCAAGCTGGCCCTGCTCATCAACCAGGTCGGCCTGTTCCTGATCGTGTCCAGCGCTGCCTGCTGTGGCATCGTCATGTTTGTGTTCTACACTGACTGCGACCCTCTCCTCCTGGGGCGCATCTCTGCCCCAGACCAGTACATGCCTCTGCTGGTGCTGGACATCTTCGAAGATCTGCCTGGAGTCCCCGGGCTTTTCCTGGCCTGTGCTTACAGTGGCACCCTCAGCACAGCATCCACCAGCATCAATGCTATGGCTGCAGTCACTGTAGAAGACCTCATCAAACCTCGGCTGCGGAGCCTGGCACCCAGGAAACTCGTGATTATCTCCAAGGGGCTCTCACTCATCTACGGATCGGCCTGTCTCACCGTGGCAGCCCTGTCCTCACTGCTCGGAGGAGGTGTCCTTCAGGGCTCCTTCACCGTCATGGGAGTCATCAGCGGCCCCCTGCTGGGAGCCTTCATCTTGGGAATGTTCCTGCCGGCCTGCAACACACCGGGCGTCCTCGCGGGACTAGGCGCGGGCTTGGCGCTGTCGCTGTGGGTGGCCTTGGGCGCCACGCTGTACCCACCCAGCGAGCAGACCATGAGGGTCCTGCCATCGTCGGCTGCCCGCTGCGTGGCTCTCTCAGTCAACGCCTCTGGCCTCCTGGACCCGGCTCTCCTCCCTGCTAACGACTCCAGCAGGGCCCCCAGCTCAGGAATGGACGCCAGCCGACCCGCCTTAGCTGACAGCTTCTATGCCATCTCCTATCTCTATTACGGTGCCCTGGGCACGCTGACCACTGTGCTGTGCGGAGCCCTCATCAGCTGCCTGACAGGCCCCACCAAGCGCAGCACCCTGGCCCCGGGATTGTTGTGGTGGGACCTCGCACGGCAGACAGCATCAGTGGCCCCCAAGGAAGAAGTGGCCATCCTGGATGACAACTTGGTCAAGGGTCCTGAAGAACTCCCCACTGGAAACAAGAAGCCCCCTGGCTTCCTGCCCACCAATGAGGATCGTCTGTTTTTCTTGGGGCAGAAGGAGCTGGAGGGGGCTGGCTCTTGGACCCCTTGTGTTGGACATGATGGTGGTCGAGACCAGCAGGAGACAAACCTC**GGCATTCTGCAGTCcACGGTtCCGCGcGCCCGGGATCCACCGGTCGCCACCATGGTGTCTAAGGGCGAAGAGCTGATTAAGGAGAACATGCACATGAAGCTGTACATGGAGGGCACCGTGAACAACCACCACTTCAAGTGCACATCCGAGGGCGAAGGCAAGCCCTACGAGGGCACCCAGACCATGAGAATCAAGGTGGTCGAGGGCGGCCCTCTCCCCTTCGCCTTCGACATCCTGGCTACCAGCTTCATGTACGGCAGCAGAACCTTCATCAACCACACCCAGGGCATCCCCGACTTCTTTAAGCAGTCCTTCCCTGAGGGCTTCACATGGGAGAGAGTCACCACATACGAAGACGGGGGCGTGCTGACCGCTACCCAGGACACCAGCCTCCAGGACGGCTGCCTCATCTACAACGTCAAGATCAGAGGGGTGAACTTCCCATCCAACGGCCCTGTGATGCAGAAGAAAACACTCGGCTGGGAGGCCAACACCGAGATGCTGTACCCCGCTGACGGCGGCCTGGAAGGCAGAAGCGACATGGCCCTGAAGCTCGTGGGCGGGGGCCACCTGATCTGCAACTTCAAGACCACATACAGATCCAAGAAACCCGCTAAGAACCTCAAGATGCCCGGCGTCTACTATGTGGACCACAGACTGGAAAGAATCAAGGAGGCCGACAAAGAGACCTACGTCGAGCAGCACGAGGTGGCTGTGGCCAGATACTGCGACCTCCCTAGCAAACTGGGGCACAAACTTAATTGA.

**
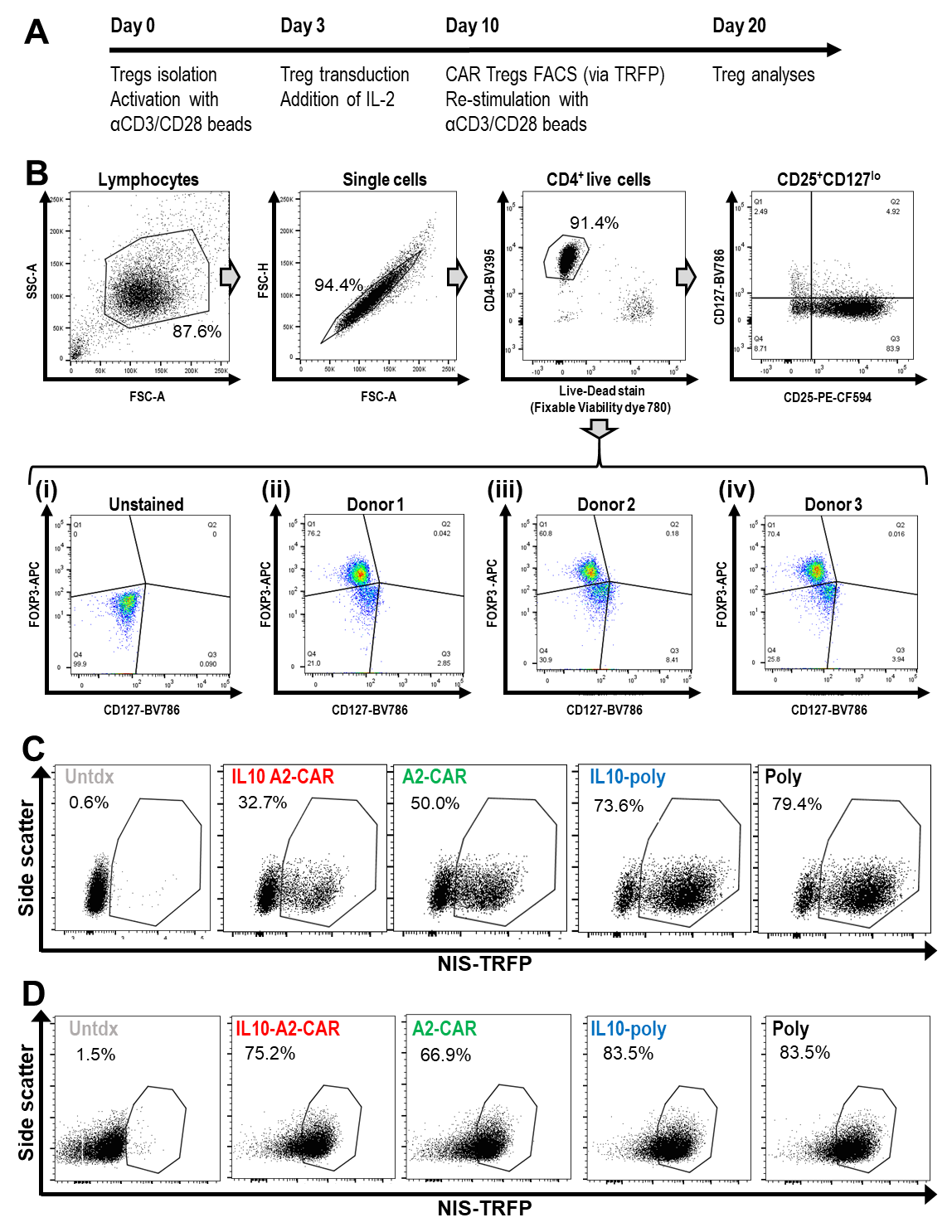
**

**Supplementary Fig.S1. Additional information to CAR-Treg generation | (A)**Scheme for CAR-Treg production. **(B)**: CD4^+^T cells were isolated from total peripheral blood monocytes by negative selection on CD4 using Rosette-Sep^TM^ and CD4^+^T cells were enriched for CD25^+^T cells using CD25 Microbeads II. The purity of these Treg population was assessed by flow cytometry with gating as indicated.in the *upper panel*. The predominant population was CD4^+^CD25^+^CD127^lo^. Analysis of FOXP3 alongside CD127 is shown in the *lower panel* and demonstrated, for three different donors, the quality of Tregs obtained. **(i):** unstained control (**ii-iv)** staining on three different representative donors. Mean percentage of FoxP3^+^ and CD127^lo^ cells here were 69.1%. These cells were used as input for CAR-Treg generation. **(C)** Representative flow cytometry data of transduced Tregs determined 10 days after transduction and prior to FACS sorting. **(D)** Representative flow cytometry data of sorted and further expanded Tregs (day 20). Corresponding cumulative data are shown in Fig.1B.

**Supplementary Fig.S2. Assessment of radionuclide reporter function |** Indicated Tregs were cultured with 50kBq of the NIS radiotracer [^99m^Tc]TcO_4_^-^ for 30 minutes. The radiotracer was used at pM concentrations and thus far below the Michaelis-Menten constant of NIS (mM range) and hence at concentrations that are proportional to the NIS uptake rate. Shown is the amount of radiotracer taken up by the cells in percent of the total amount of radiotracer in the assay. Notably, the radiotracer was present in large excess for the given assay conditions with NIS-TRFP expressing cells still taking up ~15-20% of the total radiotracer amount. In the presence of ClO_4_^‑^, a competing NIS substrate, [^99m^Tc]TcO_4_^-^ uptake of NIS-TRFP expressing cells was significantly reduced. Together this demonstrated NIS-TRFP function and specificity in these Treg types. Cumulative radiotracer uptake data of *n*=6 different Treg batches each. Analysis by 1‑way ANOVA with Tukey’s multiple comparison correction; error bars are SEM.

**
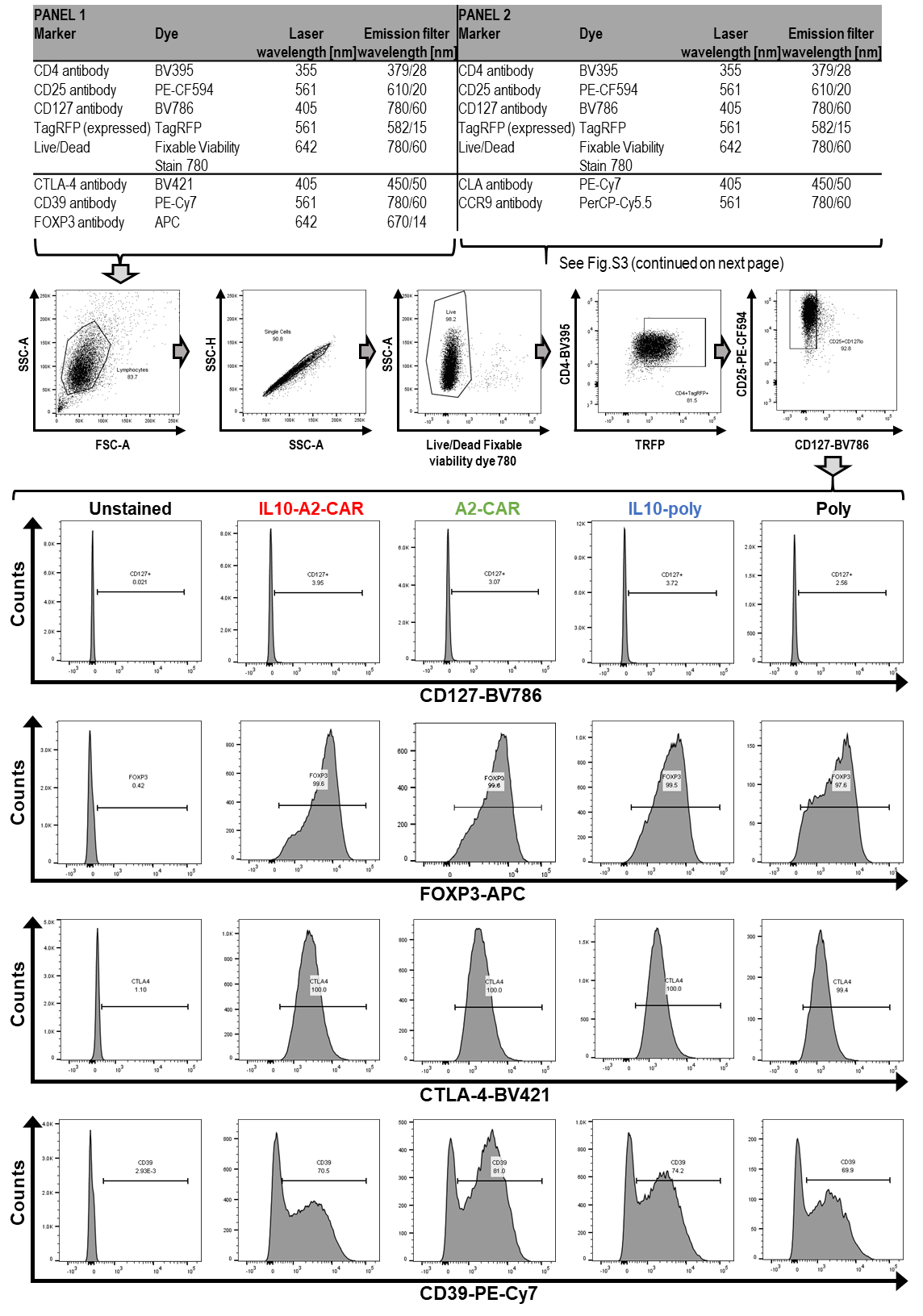
**

**Supplementary Fig.S3. Additional data supporting phenotypic marker analyses |** Representative histograms of indicated flow cytometric marker analyses corresponding to data shown in Fig.2B and the chemokines from Fig.2C; including staining/marker panels and gating strategy. (Continued on next page).


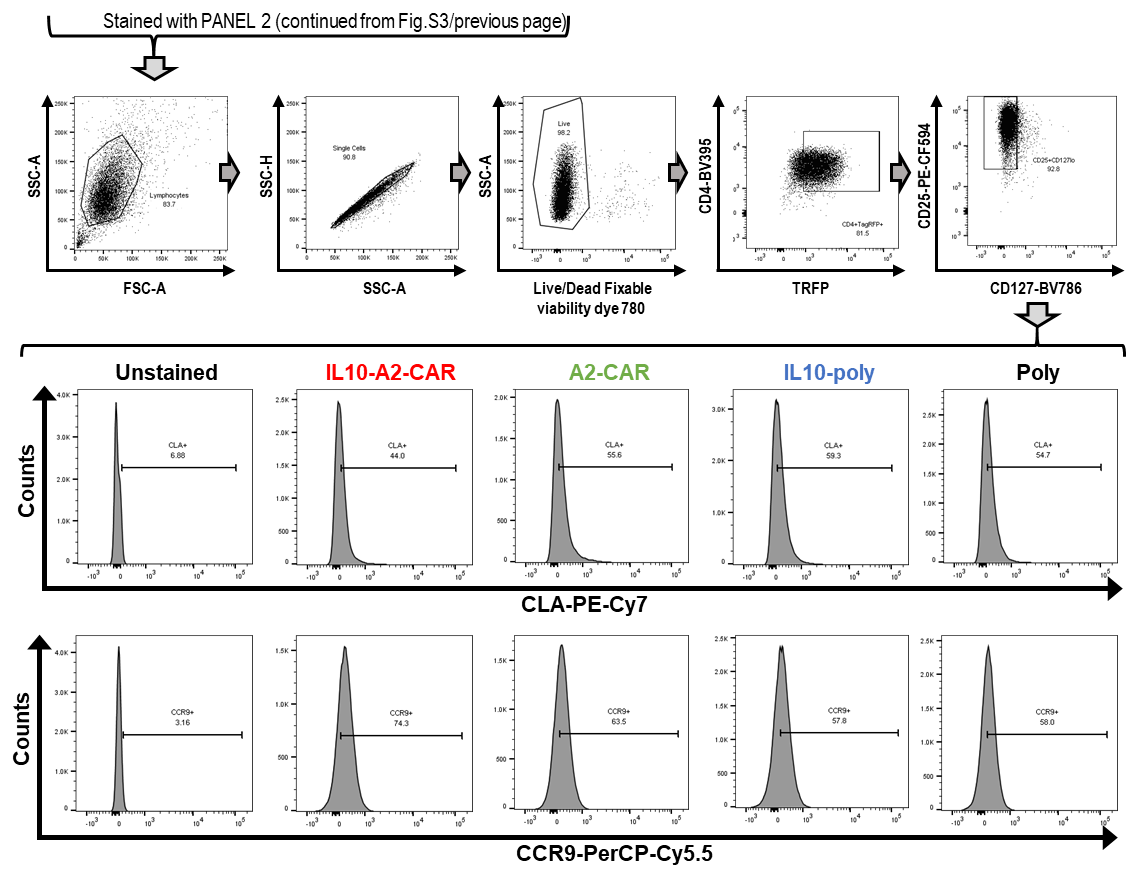


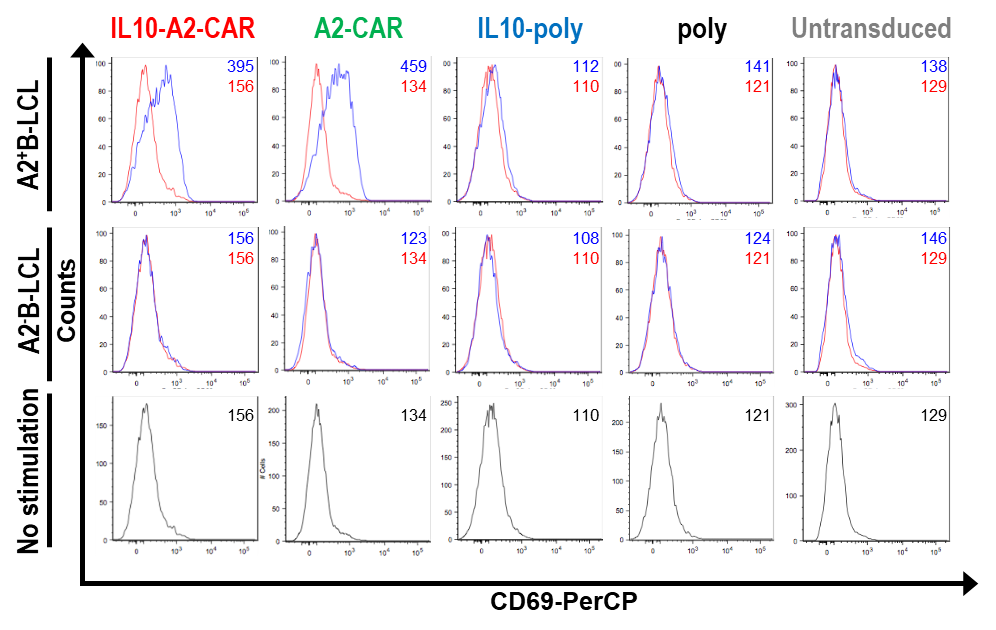


**Supplementary Fig.S4. Antigen-specific Treg activation |** Indicated Tregs were co-cultured for 18h with indicated B-LCLs or remained unstimulated for this period. Subsequently, Tregs were harvested, immunostained with an anti-CD69 antibody and analysed by flow cytometry. Representative histograms are shown supplementing the cumulative data in Fig.3A. Stimulated cells are compared to unstimulated cells (top two rows: *stimulated = blue*, *unstimulated = red*; bottom row unstimulated cells only). Values are mean fluorescence intensity (MFI).

**
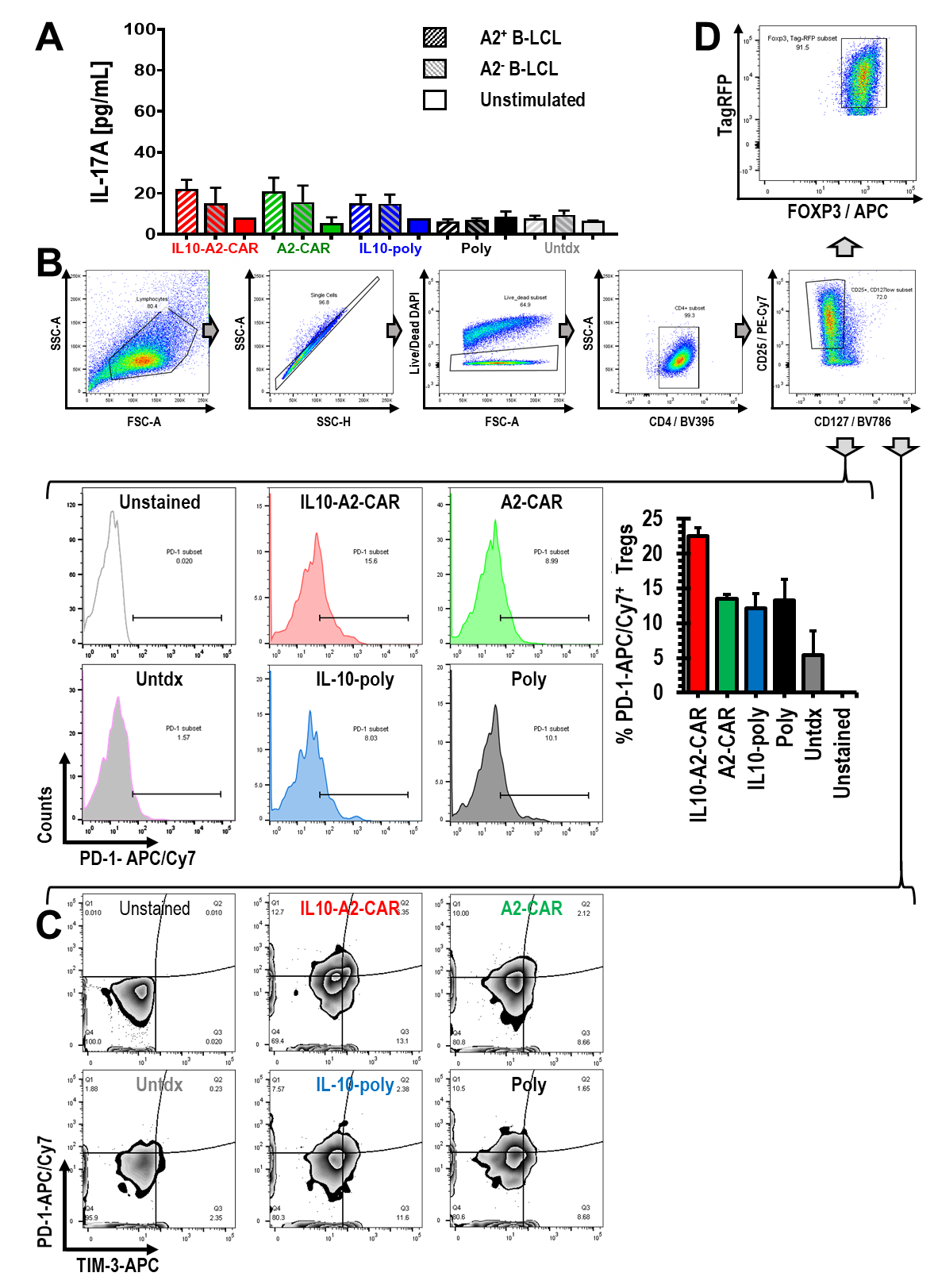
**

**Supplementary Fig.S5. Engineered Tregs produce little IL-17A and only few express little amounts of PD-1 | (A)** Culture supernatants from CD69 activation assays were collected and analysed for secreted IL-17A using a cytokine bead array. Error bars represent SEM from *n=*8 Treg batches/donors. No significant differences between A2^+^ B-LCL or A2^-^ B-LCL stimulated Tregs were found*.* The overall levels detected were very low across all cell and treatment types compared to published values CD28-signalling domain-based CAR-Tregs and effector T cells under similar conditions (ranging 200-1000 pg/mL; Ref 22). **(B/left)** Indicated Tregs were immunostained including an anti-PD-1 antibody. Representative histograms of flow cytometric analysis of PD-1 after gating on live CD4^+^CD25^+^CD127^lo^TRFP^+^ Tregs; grey-shaded area represent region of interest for PD-1^+^ cells. All Treg types stemmed from the same donor in this example. **(B/right)** Cumulative data of Treg types from *n*=2 different donors with error bars representing SD. **(C)** Live CD4^+^CD25^+^CD127^lo^TRFP^+^ Tregs from (B/left) were also immunostained for Tim-3 indicating that not only few engineered Tregs were positive for both markers.

**
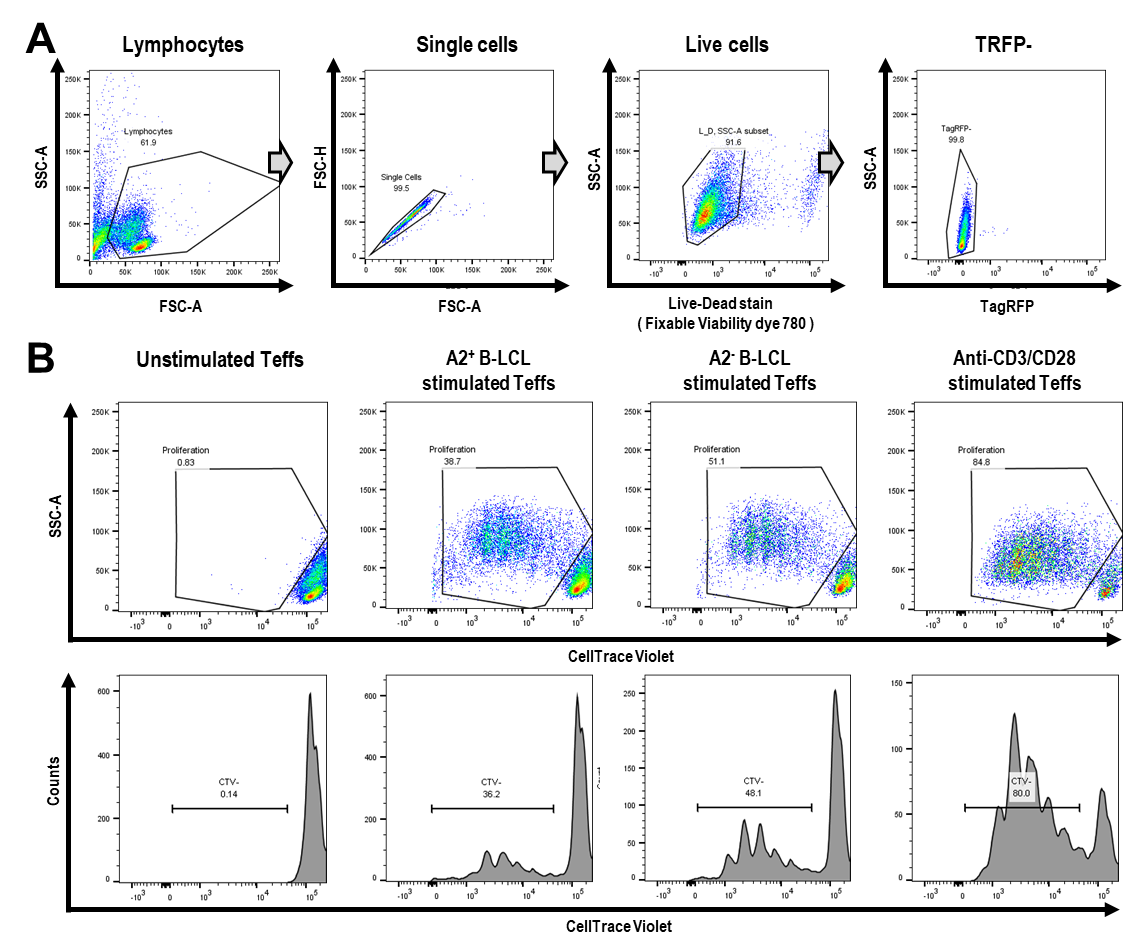
**

**Supplementary Fig.S6. Gating and controls for the quantification of Teff proliferation accompanying Treg suppression assays. | (A)** Teffs were gated on lymphocytes, single cells, live cells and being negative for the NIS-TRFP reporter. **(B)** Subsequently, Teffs were identified as through their labelling with Cell Trace Violet (CTV). Unstimulated Teffs did not proliferate, while stimulated Teffs proliferated; the latter being quantified from the label loss caused by each Teff cell division. A2^+^ B-LCL, A2^-^ B-LCL and anti-CD3/CD28 beads all stimulated Teffs. Representative experiments that served as controls and to determine maximum proliferation for assays to quantify suppressive capacity of Tregs.

**
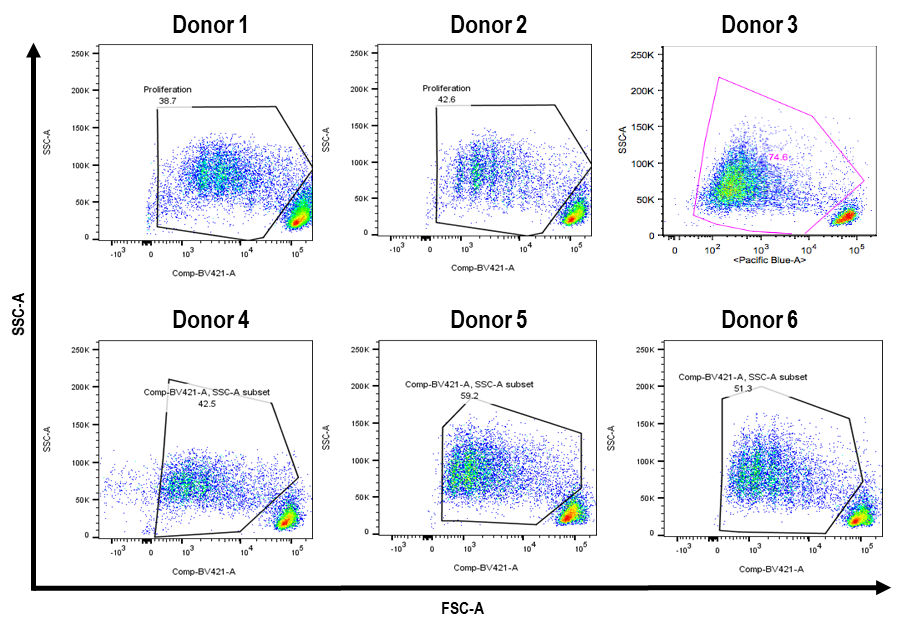
**

**Supplementary Fig.S7. Teff suppression by A2^+^ B-LCL for different donors. |** Experiments demonstrating that Teffs of each donor in this study proliferated upon stimulation with A2+ B‑LCL and in the absence of Tregs. These experiments provided the relevant maximum proliferation values required for the determination of the suppressive capabilities of various Treg types (*cf.* Fig.3).


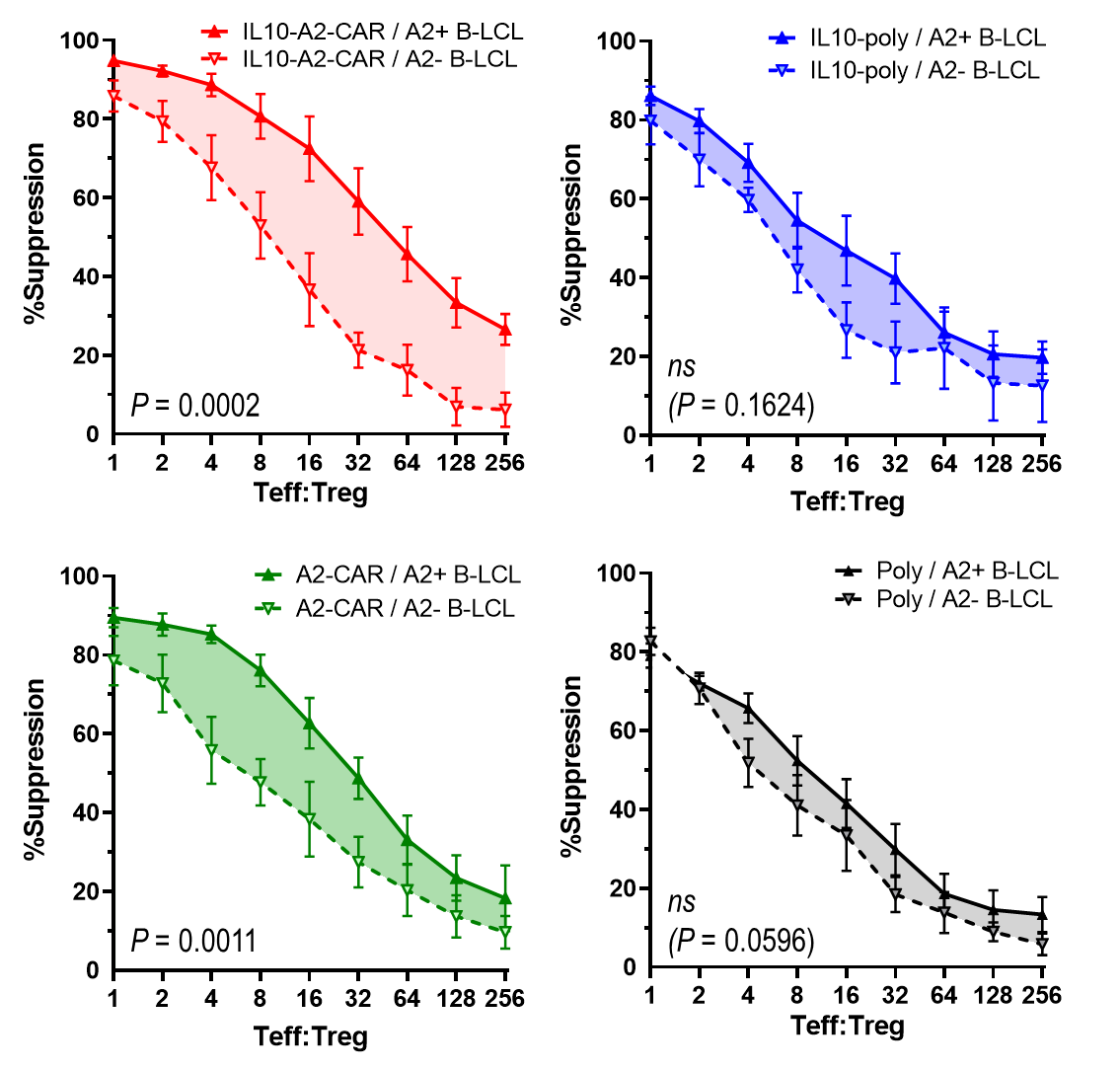


**Supplementary Fig.S8. Suppression assay data from Fig.3 but visualized for the comparison of suppressive capacity in the presence of A2^+^ versus A2^-^ B-LCLs |** The colour scheme is aligned with all other figures and Treg types indicated in the respective inset legends. Data are from *n*=6 different Treg batches. Statistical analysis was by Mixed-model 2-way ANOVA with matched pairs per donor batch with *p*-values added to figure panels. Error bars are SEM.

**
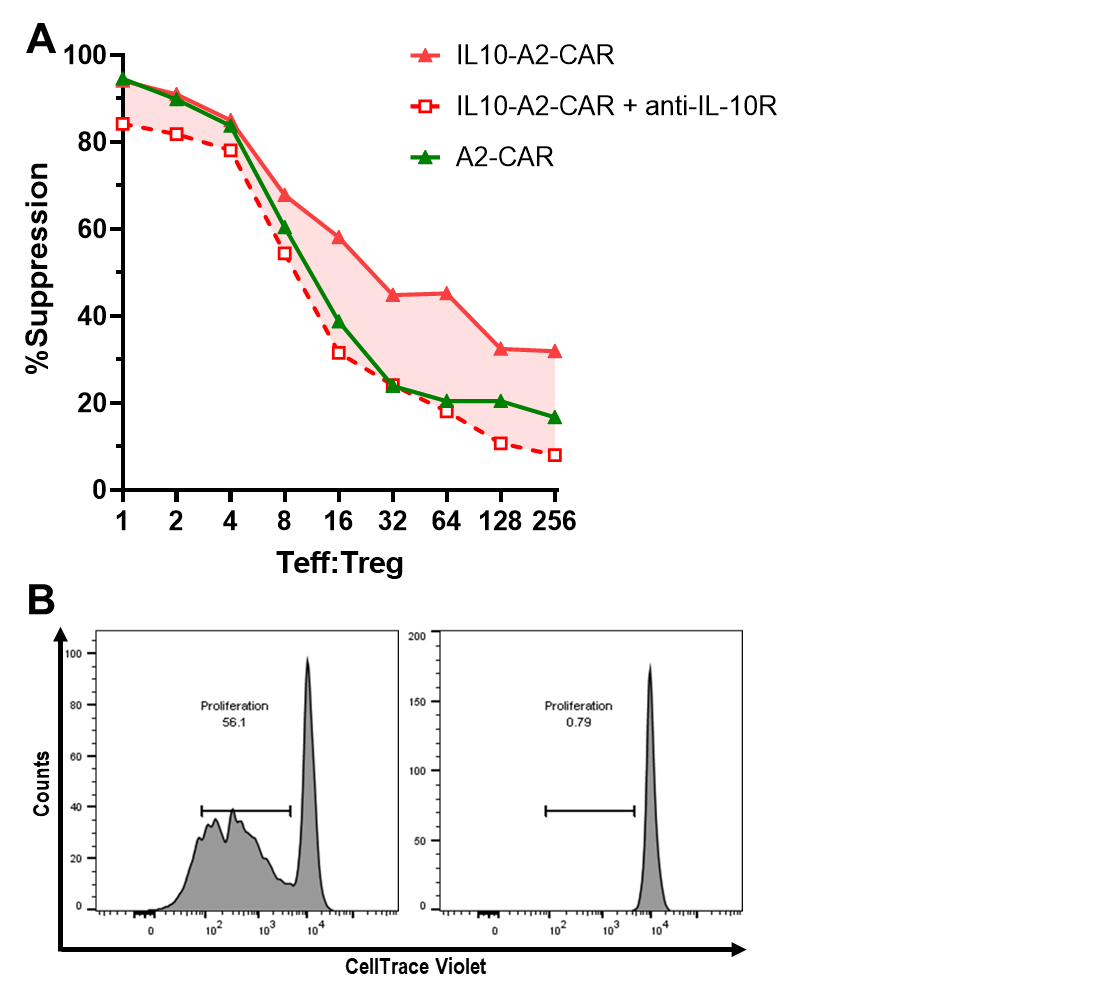
**

**Supplementary Fig.S9. Suppressive capacity of IL10-A2-CAR Tregs in the presence of an IL-10 receptor blocking antibody |** Suppression assays were performed with IL10-A2-CAR Tregs essentially as in Fig.3E-F but either in the presence or absence of a monoclonal IL-10 receptor blocking antibody (clone 3F9; 15 μg/mL). A2-CAR Tregs served as additional controls in this experiment. **(A)** Indicated Treg types with or without IL-10R blockade. Cells were co-cultured with CellTrace Violet-labelled Teffs and A2+ B-LCL for five days at indicated Teff:Treg ratios (cf. Materials and Methods). Suppression of Teff proliferation was measured by Teff label dilution using flow cytometry. Shown is the suppression of Teff proliferation by Tregs stimulated with either A2+ B-LCL for the indicated conditions. One representative dataset is shown in which all Treg types were made from the same donor and Teffs were autologous to Tregs. **(B)** Corresponding Teff proliferation controls. (Left) Stimulation with A2+ B-LCL resulted in proliferating Teffs did proliferate while (right) unstimulated Teffs did not proliferate. Representative experiments are shown, which served as controls and to determine maximum proliferation for assays to quantify suppressive capacity of Tregs.

**
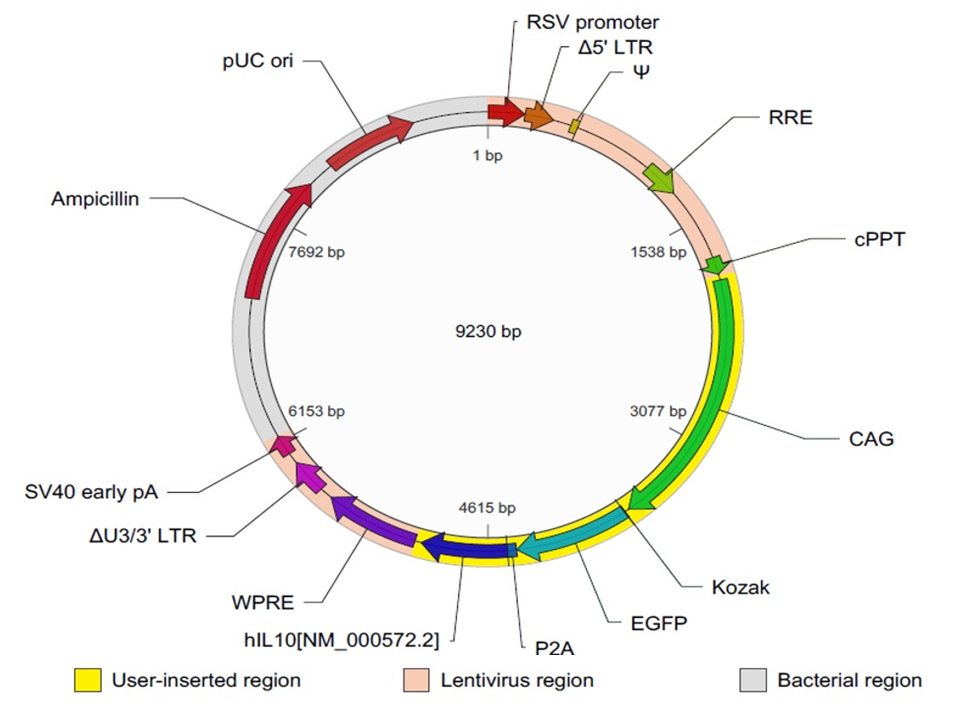
**

**Supplementary Fig.S10. Source of the human IL-10 used in this work |** A lentiviral vector carrying an expression cassette for human IL-10 and various other components. The source material as outlines here as well as this map were kindly gifted by Dr Kate Milward. The corresponding sequence is highlighted in Supplementary Tabs.S3 and S4, respectively.

**
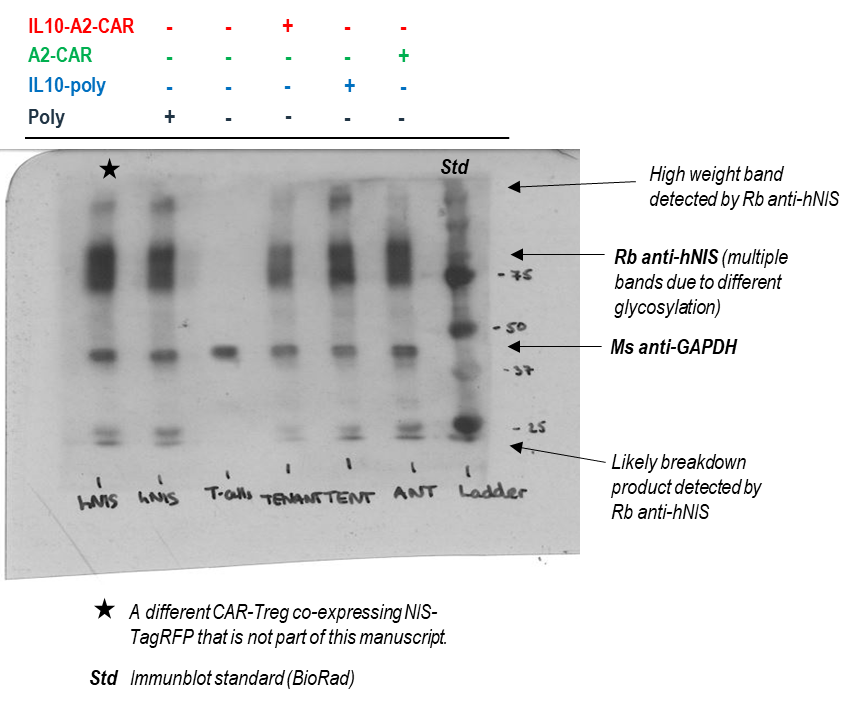
**

**Supplementary Fig.S11.** Full view of double-probed immunoblot shown in Fig.1C including relevant annotations.

***** End of Supporting Information *****
